# Machine learning-based analytics of the impact of the Covid-19 pandemic on alcohol consumption habit changes among United States healthcare workers

**DOI:** 10.1038/s41598-023-33222-y

**Published:** 2023-04-12

**Authors:** Mostafa Rezapour, Muhammad Khalid Khan Niazi, Metin Nafi Gurcan

**Affiliations:** Center for Biomedical Informatics, Wake Forest University School of Medicine, Winston-Salem, NC USA

**Keywords:** Diseases, Health care, Mathematics and computing

## Abstract

The COVID-19 pandemic is a global health concern that has spread around the globe. Machine Learning is promising in the fight against the COVID-19 pandemic. Machine learning and artificial intelligence have been employed by various healthcare providers, scientists, and clinicians in medical industries in the fight against COVID-19 disease. In this paper, we discuss the impact of the Covid-19 pandemic on alcohol consumption habit changes among healthcare workers in the United States during the first wave of the Covid-19 pandemic. We utilize multiple supervised and unsupervised machine learning methods and models such as decision trees, logistic regression, support vector machines, multilayer perceptron, XGBoost, CatBoost, LightGBM, AdaBoost, Chi-Squared Test, mutual information, KModes clustering and the synthetic minority oversampling technique on a mental health survey data obtained from the University of Michigan Inter-University Consortium for Political and Social Research to investigate the links between COVID-19-related deleterious effects and changes in alcohol consumption habits among healthcare workers. Through the interpretation of the supervised and unsupervised methods, we have concluded that healthcare workers whose children stayed home during the first wave in the US consumed more alcohol. We also found that the work schedule changes due to the Covid-19 pandemic led to a change in alcohol use habits. Changes in food consumption, age, gender, geographical characteristics, changes in sleep habits, the amount of news consumption, and screen time are also important predictors of an increase in alcohol use among healthcare workers in the United States.

## Introduction

COVID-19, caused by a virus named SARS-CoV-2, was first discovered in December 2019 in Wuhan, China^1^. The Covid-19 pandemic caused an unprecedented health crisis worldwide and negatively affected the mental health of healthcare workers^2–4^. A rise in the rate of positive COVID-19 tests and the number of hospitalizations, reduction of proper personal protection equipment, working under severe pressures, and an increase in fears related to contracting the virus and transmitting it to others could contribute to a mental health decline among healthcare workers^5–7^. Many research articles have studied the impact of the Covid-19 pandemic on the mental health decline of healthcare staff^8–11^. Previous disasters, such as 9/11 and Hurricane Katrina, have demonstrated that the stress of the events, as well as anxiety about the future, can increase drinking and aggravate alcohol use disorder symptoms. Excessive alcohol intake may influence not only COVID-19 susceptibility and severity, but the pandemic's overall consequences are likely to add to excessive alcohol consumption. As part of this study, we investigate the relationships between COVID-19-related negative effects and alterations in the drinking patterns of healthcare professionals. But first, we will quickly discuss how machine learning has been utilized to solve Covid-related challenges. Many approaches exist for diagnosing and treating COVID-19, including DNA sequencing and CT scans. However, because of the exponential rise in COVID-19 cases during the first wave of the pandemic, traditional diagnostic techniques became ineffective. Machine learning and deep learning have holden valuable tools that can revolutionize the processes of forecasting, screening, and detecting COVID-19.

- **Social impacts of the pandemic:**

In June 2020, Beiter et al.^12^ conducted an anonymous cross-sectional survey through email with around 550 healthcare workers at an academic hospital in New Orleans, Louisiana. The survey had five sections but only the first two were analyzed, which measured work-related stress, emotional responses to the pandemic, depressive symptoms, coping habits, and pre- and current COVID-19 alcohol consumption. The study found that the pandemic has increased stress among healthcare workers, with around half of them reporting high alcohol consumption. The predictors of higher alcohol consumption included being White and using avoidant coping, while being single, having depression symptoms, and having a strong emotional reaction to the pandemic were predictors of a greater increase in alcohol consumption. The study also noted the potential for social desirability bias and the risk of normalizing increased alcohol consumption among healthcare workers during pandemics.

Mongeau-Pérusse et al.^13^ conducted a survey to explore the effects of COVID-19 confinement on various outcomes such as mental health, social interactions, and quality of life. The study collected data on personal characteristics, employment status, health conditions, stress levels, and alcohol use behavior. Participants were recruited between May 25 and June 26, 2020, from online advertising on social media platforms. To be eligible, participants had to be employed, 18 years or older, and residing in Quebec. The study included 847 participants out of 1165 adults who accessed the survey. A pre-test was conducted in France on a group of 40 participants to validate the survey questions, and a separate questionnaire was adapted for Quebec participants. All participants provided electronic consent before completing the survey, and the study received ethical approval from the Hautes Études Commerciales de Montréal. The study found that daily alcohol consumption and alcohol craving increased during the confinement with a small to medium effect size. Alcohol supply sources remained open in Quebec during the pandemic, which may have contributed to the increase in alcohol consumption. The study's results are consistent with alcohol consumption levels in other populations worldwide, and several factors could explain the increase in alcohol consumption during the confinement, including prolonged social isolation, psychosocial stressors, and the use of alcohol as a self-medication for anxiety, stress, and worry related to the pandemic. The study's objective was to examine how the COVID-19 pandemic influenced the drinking habits of Quebec's working population. The study revealed that there was a rise in both daily alcohol consumption and alcohol craving during the confinement period, with a moderate effect size, as alcohol supply sources remained open in Quebec. The researchers found no disparity in alcohol consumption between healthcare and non-healthcare workers^13^.

In a recently published paper, Pomazal et al.^14^ aimed to identify factors related to changes in alcohol consumption during the COVID-19 pandemic in Wisconsin. Three surveys were conducted at different time points during the first 18 months of the pandemic among past participants of the Survey of the Health of Wisconsin. The analyses were conducted in SAS v9.4. The participants were categorized based on their alcohol consumption responses, and those who completed the questions related to alcohol consumption were included in the analysis. Chi-squared tests were used to compare within-survey univariate differences, and logistic regression modeling was completed to model odds of increased drinking. The sample was restricted to those between 21 and 60 years of age and stratified by the presence of children in the home. The study found that younger age, anxiety, higher education levels, higher income, working remotely, and having children at home were all significantly associated with increased drinking in all three waves. Younger age was identified as the most important predictor of increased alcohol consumption at all three time points, indicating that young adults in Wisconsin may be at higher risk for heavy drinking during the pandemic. The study found that reports of increased drinking slightly decreased in each wave, and those reporting drinking as about the same increased in each wave.

During the COVID-19 pandemic in Brazil, Mota et al.^15^ conducted an observational and cross-sectional study from May to July 2020. The study was approved by the Federal University of Paraiba's ethics committee and the National Commission for Ethics and Research. A Google Forms questionnaire was distributed to healthcare professionals across Brazil through online groups on WhatsApp and the Brazilian Hospital Services Company's website. Participants had to consent to the Free and Informed Consent Term to ensure confidentiality and anonymity. The inclusion criteria were healthcare professionals working during the pandemic, and there were no exclusion criteria. The study had 710 participants, mostly female physicians from Paraíba, Brazil. Around two-thirds of the participants reported having sleep-related complaints, with a significant proportion using insomnia medication, mostly through self-medication. Many participants also reported changes in their diet and physical activity, with a significant proportion increasing their alcohol consumption. The study concludes that the impact of COVID-19 on the quality of life of healthcare professionals in Brazil was higher than what has been reported in international surveys for the general population.

Calina et al.^16^ discuss the impact of alcohol consumption during the COVID-19 pandemic. They caution that alcohol consumption during social distancing may lead to physical and mental health issues, especially for those with pre-existing conditions. Alcohol consumption can also weaken the immune system and contribute to obesity, as well as encourage risky behavior, such as aggression and depression, which can increase the risk of contracting COVID-19. They emphasize the importance of regular drinkers re-evaluating their alcohol intake during the pandemic, as excessive alcohol consumption is linked to millions of premature deaths globally each year. The pandemic may also have long-term effects on health behavior and status, including increased acceptance of alcohol use in families and greater consumption by pregnant women, leading to fetal alcohol spectrum disorders (FASD). The article suggests conducting a detailed analysis of the root causes and interconnections between alcohol use, mental health, socio-economic conditions, and social support to develop targeted public policies that focus on the groups most susceptible to harmful alcohol consumption.

Saladino et al.^17^ bring together finding from several other studies to provide an overview of the numerous psychological and social effects that are brought forth through quarantine. During the Covid-19 pandemic, social distancing and the fear of contracting the disease led to an increase in stress within social groups and have made people more likely to develop various symptoms of distress such as Posttraumatic Stress Disorder (PTSD) and anxiety^17^. Separation from loved ones and friends, a feeling of helplessness, and a feeling locked up are all reasons for the increased psychological stress brought forth by the pandemic. When a family member or friend is infected with Covid-19, there are worsening anxiety symptoms and higher levels of stress. Also, when schools or academic institutions are closed due to the pandemic, there is a notable anxiety increase during these times^17^.

Moreover, due to the lack of healthcare workers during the pandemic, healthcare workers were put under increased levels of stress in their respective jobs. Healthcare workers were at higher chances of developing symptoms such as PTSD, burnout syndrome, and depersonalization. As a result of the Covid-19 pandemic, many healthcare workers were overworked and put under high pressure at their jobs. This led to higher levels of psychophysical stress and secondary traumatic stress disorder^17^.

Mireia et al.^18^ surveyed the stress levels of the parents, perceived stress levels of the children, and the overall family’s well-being. In their study, 1,143 participants were surveyed in numerous cities throughout Italy and Spain. The study's respondents were from 18 to 66 and all were primary caregivers to children. The study recruited individuals through social media and administered the study through a google form sent to their inboxes. They have concluded that 85% of parents observed changes in their children’s behavior and overall emotional state due to the implementation of quarantine. There were several increases in certain behaviors and emotions such as higher difficulty concentration, increased boredom, increased nervousness, and increased irritability. These differences show the effects Covid-19 has on every household away from the workplace and even effects children who had to quarantine.

Brooks et al.^19^ found 3166 papers researched about how quarantine can affect the social well-being and mental health of individuals. Of the 3166 papers found, 24 papers were chosen from various quarantines imposed by different diseases such as Covid-19, SARS, and Ebola. According to their research, quarantine can enhance emotions such as anger and sadness and oftentimes lead to feelings of loneliness^19^. In a study researching the SARS pandemic, Bai et al.^20^ found that during the quarantine period, healthcare workers who had previously been quarantined were at the highest risk of developing acute stress disorder.

Lai et al.^21^ studied the factors that are associated with mental health among healthcare workers in China. In this study, 1257 healthcare workers across 34 hospitals were analyzed about their mental health status. These healthcare workers all dealt with Covid-19 patients during the onset of the Covid-19 pandemic. The data was collected at the end of January in 2020. The 9-item Patient Health Questionnaire, 7-item Generalized Anxiety Disorder scale, 7-item insomnia severity index and the 22-item Impact of Event Scale were used to gather data concerning the anxiety and depression levels in healthcare workers. Demographic data were recorded as well, with questions describing the sex of the respondent along with contributing factors such as age, education level, and marital status. Their results show that 50 percent of respondents reported symptoms of depression, along with 44.6 percent reporting symptoms of anxiety. Insomnia was reported at 34 percent, and 71% of healthcare workers reported feeling distressed from their work. Nurses and front-line workers were found to have more severe symptoms of depression, anxiety, and insomnia than other hospital workers. Healthcare workers working in primary and secondary hospitals, which are hospitals dealing with infected Covid-19 patients, were found to display more severe symptoms of depression and anxiety. Lai et al.^21^ noticed the difference in the severity of symptoms between male and female healthcare workers. Female workers tended to display more severe symptoms of depression and anxiety than their male counterparts.

De Kock et al.^22^ studied the effect of the Covid-19 pandemic on healthcare workers. They reviewed the findings of other studies to determine if there exist high-risk factors for healthcare workers during the pandemic. They found that healthcare workers during the Covid-19 pandemic had higher levels of depression, anxiety, and obsessive–compulsive disorder (OCD). Moreover, they found that medical healthcare workers, which consist of nurses and doctors, had significantly higher mental health risks than non-medical healthcare workers. Also, the staff that was directly dealing with patients reported higher levels of stress, depression, and somatization. Another factor affecting the healthcare workers was the workload placed on them during this time. Longer working hours were associated with a higher risk of developing these factors. Younger aged workers and female workers were discovered to develop worse mental health symptoms than those of their older and male counterparts. Along those lines, most nurses dealing on the front line with patients were younger-aged females, which could lead to some of discrepancy^22^.

- **Machine learning methods for solving some Covid-related problems:**

Machine Learning (ML) has started playing an important role in the detection and diagnosis of COVID-19^23^. Machine learning methods have been utilized to predict the future spread of Covid-19 through logistic regression models^24^, support vector regression^25^, polynomial regression^26^, and various other models^27^. Additionally, other machine learning models such as multilayer perceptron and XGBoost are employed to tackle the problems of classifying Covid-19 patients into groups with a higher risk of death. Another influential aspect of machine learning has been altering how we diagnose patients for Covid-19. CT scans and X-Ray images are among the two most common ways of identifying Covid-19 through imaging. Deep learning methods and the use of neural networks have allowed algorithms to extract specific features from an image to predict the diagnosis. Mondal et al.^28^ compared the results of 67 papers written about the impacts of the machine and deep learning on Covid-19 and compared the results of the deep learning models. For X-Ray images, the results concluded that ResNet18 provided the most accurate results with 100% accuracy [Reference] followed by DenseNet201, which had an accuracy of 99.70% [Reference]. For CT scans, DenseNet169 produced the most accurate results with 99.80% accuracy, followed by Inception ResNet v2, having an accuracy of 99.65%^28^. These specific neural networks provided the most accurate basis for predicting the diagnosis of Covid-19 patients. Therefore, these networks need to be implemented into technologies for diagnosing patients based on CT and X-Ray images to remove human error from the diagnosing process^28^.

Researchers have utilized several advanced machine learning models and algorithms to tackle various issues related to COVID-19 and to understand the pattern of its spread better. To mitigate the spread of COVID-19, it is important to predict and estimate the further spread of the disease around the globe. Gambhir et al.^29^ discuss how polynomial regression and support vector machine (SVM) algorithms can be used to predict future COVID-19 cases. Their model, using polynomial regression, successfully predicted future COVID-19 cases with an accuracy of 93% when predicting the rise of COVID-19 cases for the next 60 days. Kushwaha et al.^30^ discuss how ML algorithms can be used to understand the nature of the virus and predict the upcoming related issues. Lalmuanawma et al.^23^ review the importance of AI and ML in screening, predicting, forecasting, contact tracing, and drug development for SARS-CoV-2 and its related epidemic.

Benvenuto et al.^31^ discuss an autoregressive integrated moving average model that can predict the spread of COVID-19. Using several datasets of the COVID-19 outbreak inside and outside Wuhan, Kucharski et al.^32^ introduce a model that can explore the possible viral spread outside Wuhan. Using John Hopkins data, Peng et al. Field^18^ selected the fifteen countries that surpassed 150,000 COVID-19 cases on May 31, 2020. Using SVM, they predicted the number of confirmed cases at a time from confirmed cases in the last seven days. They used a training dataset composed of data from each country and the respective day on which they reached 100,000 cases. Then, the model was trained by looking at data from each respective day. The researchers continued this process until May 31, 2020. The researchers then used the model to predict COVID-19 cases for future days. It turned out that polynomial kernels with degrees one and two had the best least out-of-sample mean absolute error (MAE).

In contrast, the Gaussian kernel falls into overfitting in-sample data. The Gaussian kernel had the best average in-sample MAE. In addition, polynomial kernels are the most volatile surfaces, and linear kernels are the least volatile surfaces. Previous machine learning models have also found promising results when incorporating MLP and ANFIS. Zivkovic et al.^33^ have dealt with combining the concepts of machine learning and nature-inspired algorithms to improve the accuracy of the predictions. The ANFIS model merges artificial neural networks and fuzzy interference systems to create a model that can predict the spread. Combining the model with an enhanced GA allows the model to account for evolutionary factors such as natural selection and swarm. Zivkovic et al.^33^ compared GAAE-ANFIS model against other machine learning models that have proved relatively successful in predicting the spread of viruses. The models were tested against various standard regression metrics such as RMSE, MAE, MAPE, and RMSRE. Each model was trained to optimize the parameters through similar training processes and were all input with the same Covid-19 data from a WHO study in China. Each separate model was run in 30 independent trials to compare against the values or the regression metrics. The results concluded that GAAE-ANFIS proved to be the best predictor of forecasting the future of Covid-19. The RMSE, MAE, and MAPE values were the lowest of all the models for GAAE-ANFIS while still maintaining the highest value of ${\mathrm{R}}^{2}$.

Cough audio has been a newly explored technique for early identification of Covid-19. Machine learning techniques can extract certain features from cough audio to provide help in diagnosing the patient. The implementation of this technology would allow for quick diagnosis of Covid-19 while limiting contact with the patient improving safety for healthcare professionals. Virndavanam et al.^34^ analyzed 150 different cough samples from a variety of healthy and infected Covid-19 patients. A total of 65 features were extracted from the audio of each sample to obtain precise data to input into the algorithm. Three classifiers were used in determining the outcome of the data, which were Logistic Regression, SVM, and RF. From the 65 features, the 15 most dominant features were chosen using a XuniVerse model to determine the most influential features. The 15 most dominant features were then input into machine learning algorithms to classify the cough as contagious or healthy. The three classification methods were all trained with k-fold cross-validation and then tested against one another for average accuracy, recall, precision, and F1 scores. The results concluded that the SVM classifier performed most consistently and with the best performance metrics over the Logistic Regression and RF classifiers. The Random Forest classifier performed the best in accuracy while lacking in recall value which predicts that the classifier could have used more training. The Logistic Regression classifier performed the lowest across the board while still performing adequately. The accuracy percentages were above 80% which would provide insight into the precision of cough recognition for the quick diagnosis of Covid-19 to limit the spread of the virus. These results suggest new data for incorporating SVM and RF machine-learning techniques into the diagnosis of Covid-19 through cough audio.

Using Machine learning, in one of our previous works, we utilized several machine learning models to analyze the impacts the COVID-19 pandemic has had on the mental health of frontline workers in the United States^2^. In another previous work, we used machine learning methods and statistical tests to investigate the relationship between the COVID-19 vaccines and boosters and the total case count for the Coronavirus across multiple states in the USA as well as the relationship between several selected underlying health conditions with COVID-19^35^. Moreover, we have discussed the negative impacts of the Covid-19 pandemic on students’ mental health decline^36^.

To the best of our knowledge, this paper is the first of its kind to utilize advanced supervised machine learning techniques to examine the impact of the Covid-19 pandemic on alcohol consumption habits among healthcare workers in the United States during the initial wave of the outbreak. We will apply various supervised models, such as decision trees, logistic regression, support vector machines, multilayer perceptron, XGBoost, CatBoost, LightGBM, and AdaBoost to identify the underlying factors contributing to the increase in alcohol consumption among healthcare workers during the first wave of the Covid-19 pandemic. We also employ the synthetic minority oversampling technique (SMOTE) to compare the results before and after data augmentation. Moreover, we use unsupervised techniques like Chi-Squared Test and mutual information to confirm the results generated by our supervised models and to detect the root causes of the surge in alcohol intake among healthcare workers during the initial phase of the Covid-19 pandemic. We finally employ an innovative algorithm that utilizes the K-Modes clustering method to validate the results obtained from both supervised and unsupervised models, and to discover further details and factors of the surge in alcohol intake.

## Materials and methods

### Dataset availability and ethical approval

In this study, we use survey data obtained from the University of Michigan’s Inter-university Consortium for Political and Social Research (ICPSR). The data was collected by Deirdre Conroy^37,38^ from the University of Michigan Department of Psychiatry and Cathy Goldstein from University of Michigan Department of Neurology. According to the ICPSR, “The rationale for this study was to examine whether sleep, mood, and health-related behaviors might differ between healthcare workers who transitioned to conducting care from home and those who continued to report in person to their respective hospitals or healthcare facilities.” The original data contained 916 survey responses. The data was stripped of any identifying information about the respondents. Conroy et al.^37,38^ confirm that all experiments were performed in accordance with relevant guidelines and regulations (see the “Methods” Section in^37^). The survey was undertaken after clearing the approval of the University of Michigan Institutional Review Board (HUM00180147). At that point, the Qualtrics survey link was sent via email listservs that would reach large numbers of healthcare providers in^37^. It is noted that no compensation for participation was provided in^37^. Additionally, all participants have provided full and informed consent by completing the survey in^37^.

### Data preprocessing

The dataset analyzed in this paper, the COVID Isolation on Sleep and Health in Healthcare Workers data^37,38^, contains 916 rows and 64 columns. The focus is on Question 18a, which asks about changes in alcohol consumption. The dataset had 14,678 missing values, with most variables being categorical. To treat missing values, the rows with no value in Question 18a were removed, as well as unrelated columns such as StartDate, EndDate, Status, etc. Rows with too many missing values were also removed, resulting in 273 clean rows. In order to analyze the mental health dataset, which includes both categorical and ordinal variables, the first step is to convert them into numerical values. This is done through encoding techniques such as one-hot-encoding or dummy variable encoding. Several Python packages, including OneHotEncoder, LabelEncoder, and OrdinalEncoder from the sklearn preprocessing library, are used for this purpose. By doing so, the dataset is prepared for further analysis. See Supplementary Material Section 1 for the distribution of data points between the two classes of Question 18a, and further details of data preprocessing.

### Methods

In this paper, we aim to find the top predictors of alcohol consumption habit changes among healthcare workers in the U.S. during the first wave of the Covid-19 pandemic. We first examine the relationship between the target variable, Question 18a, and the rest of the variables (categorical) via unsupervised methods such Chi-squared test^39^, and mutual information^40^. However, there might be some relations between the dependent and predictors that cannot be identified via Chi-squared and mutual information.

To examine linear, nonlinear, and complicated relations between the target variable and all predictors, we first apply supervised non-ensemble models such as logistic regression (LR)^41,42^, support vector machines (SVM)^43,44^, multilayer perceptron (MLP)^45–50^, The k-nearest neighbors (KNN)^50^ and decision trees (DT)^50^. We then employ supervised ensemble models such as XGBoost^51,52^, LightGBM^53^**,** CatBoost^54^ and AdaBoost classifier^55^. In this study, to deal with imbalanced data in which there exists an unequal distribution of data points across the classes of the target variable, we also apply the Synthetic Minority Oversampling Technique (SMOTE)^56^. Finally, through a feature selection process, we then find the top predictors of accurate and robust models.

Moreover, we apply K-modes clustering^57^ to participants whose response to Question 18a is “I am drinking more alcohol” (86.1% of all data) to examine the most important common features within this cohort.

## Results

In this section, we discuss the results of unsupervised and supervised machine learning methods for finding the top predictors of alcohol consumption habit changes among healthcare workers in the U.S. during the first wave of the Covid-19 pandemic.

### Unsupervised machine learning and feature selection

To have a better understanding of the difference between the target variable and the rest of the categorical variables, as well as explore the statistical significance of the relationship between them, we now utilize Chi-squared test and mutual Information method. *Since the target variable and most predictors are categorical (nominal or ordinal), we first apply a Chi-squared test under the hypotheses*: ${H}_{0}$: the target variable, Question 18a is statistically independent of Question i, versus the alternative hypothesis ${H}_{A}$: the target variable, Question 18a, is not statistically independent of Question i, where i ∈ {2, 3, 8, 9, 10, 11, 12, 13, 14, 15, 16, 17, 18, 19, 20, 21, 22, 23, 24, 25, 26, 27, 28, 29} (categorical variables).

We first determine whether the target variable, alcohol consumption habit changes, is independent of the input variables. By a significance level of α = 0.05, it turns out that the null hypothesis H0 is rejected for Questions 11: “Are children home from school in the house?” (p-value = 0.048), Questions 15: “Have you varied your work schedule?” (p-value = 0.037), and Questions 20: “In the last month, approximately how often did you have a drink containing alcohol?” (p-value < 0.001). It is obvious that there is a significant association between Question 18a and Question 20. But an interesting result is the existence of sufficient statistical evidence that leads to rejection of hypothesis that Question 18a is independent of Question 11. It raises a question about the relationship between COVID-related school closures and alcohol consumption habit changes among healthcare workers.

We also use mutual information methods (via mutual-info-classif from sklearn.feature-selection in Python) to find the most related features to Question 18a. It turns out that Question 20: “In the last month, approximately how often did you have a drink containing alcohol?” Question 11: “Are children home from school in the house?” and Question 15: “Have you varied your work schedule?” obtain the highest scores among all features. Like Chi-squared test, the mutual information method also emphasizes that there exists a relationship between the parenting stress associated with COVID-related school closures and alcohol consumption changes. The parenting stress might contribute to an increase in alcohol consumption and a decline in the mental health of healthcare workers too.

### Supervised machine learning and feature selection

**Figure 1:**
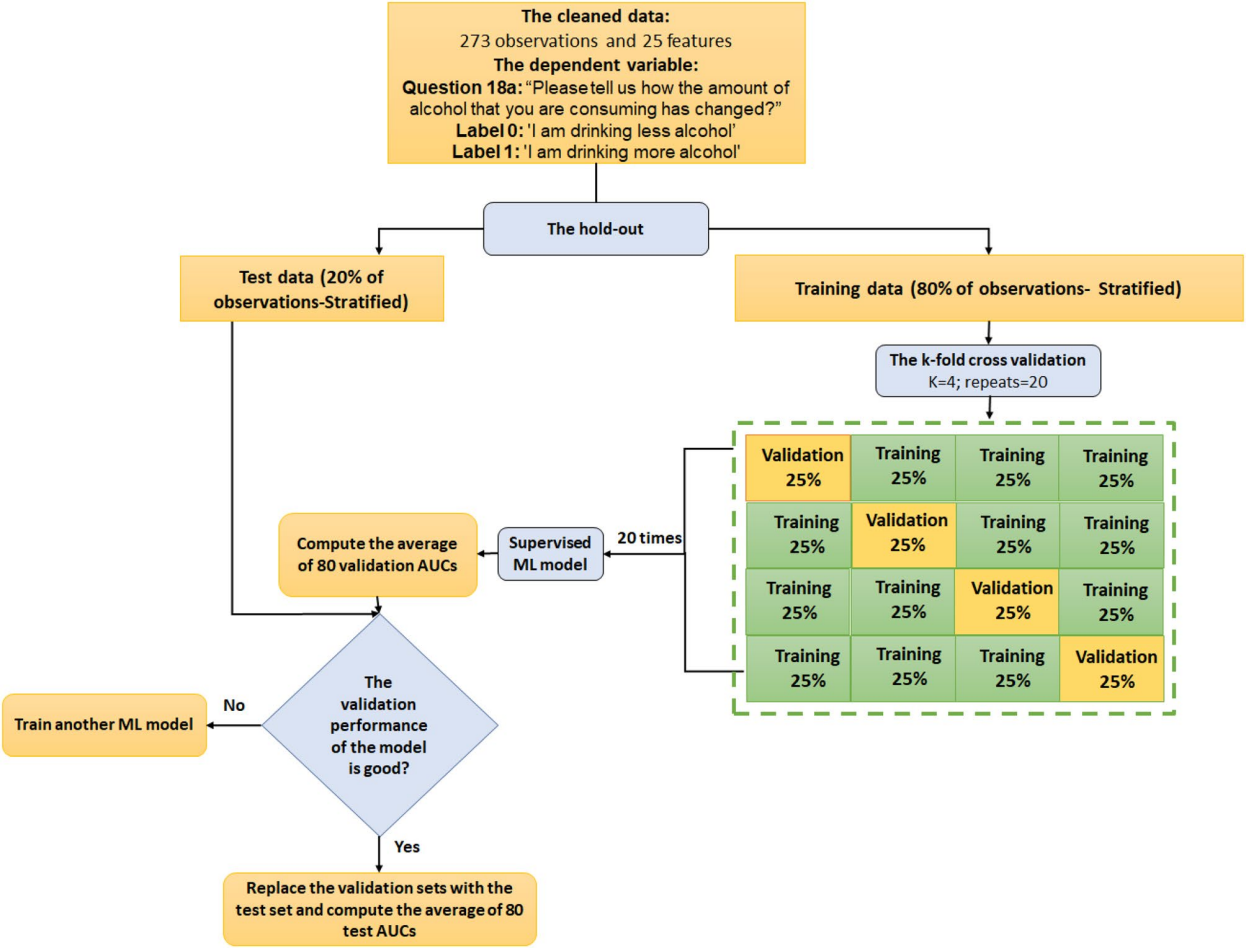
The schematic diagram of splitting the data into train, validation, and test sets.

In this section, we aim to find the most robust supervised machine learning models and analyze the importance of their features to find the top predictors of alcohol consumption habit changes among healthcare workers in the U.S. during the first wave of the Covid-19 pandemic. To train accurate models on this imbalanced data, we first apply the stratified hold-out method in which we randomly sample 20% of observations as test data, and the rest of the observations as training-validation data. To compare the performances of different ML models and tune the hyper-parameters of some models such as decision tree, random forest, XGBoost, LightGBM, and CatBoost, we apply the stratified k-fold cross validation with k = 4. We repeat the process of training-validation 20 times for each model (80 AUCs and 80 accuracy scores) and consider the average of the obtained AUCs or accuracy scores as a metric to measure the performance of a machine learning model. We then select models with a large average of validation AUCs, and an acceptable average of training AUCs (non-overfit model). Finally, we compute the average of test AUCs and accuracy scores of the selected model and analyze importance of each feature in the model prediction (feature selection) (see Fig. 1). Moreover, since the data is imbalanced, we also apply SMOTE on only training sets (neither test nor validation sets) and compare performances of models before and after SMOTE is applied to training sets.

- **Nonensemble methods**

See Supplementary Material Section 2 for details of nonensemble models such as KNN, MLP, SVM, logistic regression and decision trees with multiple depths. Table 1 displays the performances of all non-ensemble models on training, validation, and test sets before and after SMOTE is applied. Logistic regression, and decision trees perform better than other non-tree-based models, and specifically a decision tree with a maximum depth of 3 does not overfit the training dataset. Supplementary Material Fig. 2 displays the coefficients of the logistic regression when it is trained on the training data.

**Table 1:** The performance of multi-layer perceptron with two hidden layers containing 5 nodes (MLP), support vector machines (SVM), logistic regression (LR), decision tree with maximum depth of 3 (DT-3), decision tree with maximum depth of 4 (DT-4), and decision tree with maximum depth of 5 (DT-5), before and after SMOTE is applied on training dataset (SMOTE was not applied on Validation or Test sets).

Performance	Model						
	KNN (before SMOTE)	MLP (before SMOTE)	SVM (before SMOTE)	LR (before SMOTE)	DT-3 (before SMOTE)	DT-4 (before SMOTE)	DT-5 (before SMOTE)
Train AUC	0.8851	0.9658	0.8184	0.8782	0.9019	0.9767	0.9950
Validation AUC	0.6212	0.6034	0.6912	0.7217	0.7877	0.8828	0.8995
Test AUC	0.5187	0.7127	0.7771	0.8044	0.7271	0.7563	0.7900
Train accuracy	87.33%	95.92%	63.01%	91.37%	94.16%	95.13%	97.92%
Validation accuracy	85.51%	80.31%	59.03%	87.34%	91.92%	91.61%	94.02%
Test accuracy	86.58%	85.65%	63.34%	90.39%	90.41%	90.59%	93.39%

Performance	Model						
	KNN (after SMOTE)	MLP (after SMOTE)	SVM (after SMOTE)	LR (after MOTE)	DT-3 (after MOTE)	DT-4 (after MOTE)	DT-5 (after SMOTE)
Train AUC	0.9961	0.9553	0.9141	0.9496	0.9224	0.9647	0.9841
Validation AUC	0.6606	0.6327	0.6826	0.6725	0.7737	0.7820	0.8006
Test AUC	0.5056	0.7128	0.7301	0.7375	0.7807	0.8077	0.8242
Train accuracy	91.50%	91.74%	79.36%	88.38%	85.37%	91.69%	94.80%
Validation accuracy	72.08%	73.41%	71.45%	77.12%	74.06%	83.21%	85.58%
Test accuracy	62.93%	79.30%	76.30%	83.18%	76.61%	85.82%	87.82%

Decision trees perform better than other non-tree-based models, and specifically a decision tree with a maximum depth of 3 does not overfit the training dataset. A single decision tree might not be capable of exploring all features of a data, specifically when its maximum depth is restricted in order to avoid overfitting. On the other hand, tree-based ensemble models such as random forest, XGBoost, LightGBM and CatBoost can learn more details of training data without overfitting (without learning noises). In the next subsection, we apply the tree-based ensemble models.

- **Ensemble methods**

In this subsection, we explore some tree-based ensemble models including random forest, XGBoost, CatBoost and LightGBM. For all these models, we tune the numbers of trees between 2 and 10 to find the most accurate model on validation sets while it does not overfit the training set. It turns out that for all ensemble models, when the number of trees is more than 6, then they will overfit the training data. Moreover, when the number of trees is less than 4, then the models underfit the training and as a result they return small validation AUCs. Table 2 displays averages of AUCs and accuracy scores for the ensemble models before and after SMOTE is applied to training sets (neither validation nor test sets).

**Table 2:** The performance of Random Forest with 4 trees of maximum depth equals 3 (RF-4–3), Random Forest with 5 trees of maximum depth equals 3 (RF-5–3), Random Forest with 6 trees of maximum depth equals 3 (RF-6–3), XGBoostClassifier with 4 trees of maximum depth equals 3 (XGB-4–3), LightGBMClassifier with 4 trees of maximum depth equals 3 (LGBM-4–3), and CatBoostClassifier with 4 trees of maximum depth equals 3 (CAT-4–3), before and after SMOTE is applied on training dataset (SMOTE was not applied on Validation or Test sets).

Performance (averages)	Model					
	RF-4 (before SMOTE)	RF-5 (before SMOTE)	RF-6 (before SMOTE)	XGB-4 (before SMOTE)	LGBM-4 (before SMOTE)	CAT-4 (before SMOTE)
Train AUC	0.8708	0.8763	0.8831	0.9567	0.8740	0.9240
Validation AUC	0.7180	0.7189	0.7246	**0.8895**	0.7767	0.8127
Test AUC	0.7827	0.7794	0.7710	**0.8458**	0.8011	0.8973
Train accuracy	92.27%	92.07%	91.86%	92.21%	86.24%	92.72%
Validation accuracy	91.04%	90.56%	90.23%	**91.66%**	86.25%	90.88%
Test accuracy	91.27%	90.52%	90.18%	**92.57%**	85.45%	90.68%

Performance (averages)	Model					
	RF-4 (after SMOTE)	RF-5 (after SMOTE)	RF-6 (after MOTE)	XGB-4 (after MOTE)	LGBM-4 (after MOTE)	CAT-4 (after SMOTE)
Train AUC	0.9448	0.9587	0.9624	0.9767	0.9586	0.9836
Validation AUC	0.7277	0.7513	0.7605	**0.8471**	0.8299	0.8428
Test AUC	0.7447	0.7844	0.7828	**0.9109**	0.8901	0.8902
Train accuracy	87.30%	88.47%	89.16%	92.06%	89.40%	93.54%
Validation accuracy	79.07%	83.57%	82.98%	85.06%	81.95%	85.69%
Test accuracy	79.98%	84.45%	82.91%	88.61%	85.77%	89.75%

**Figure 2:**
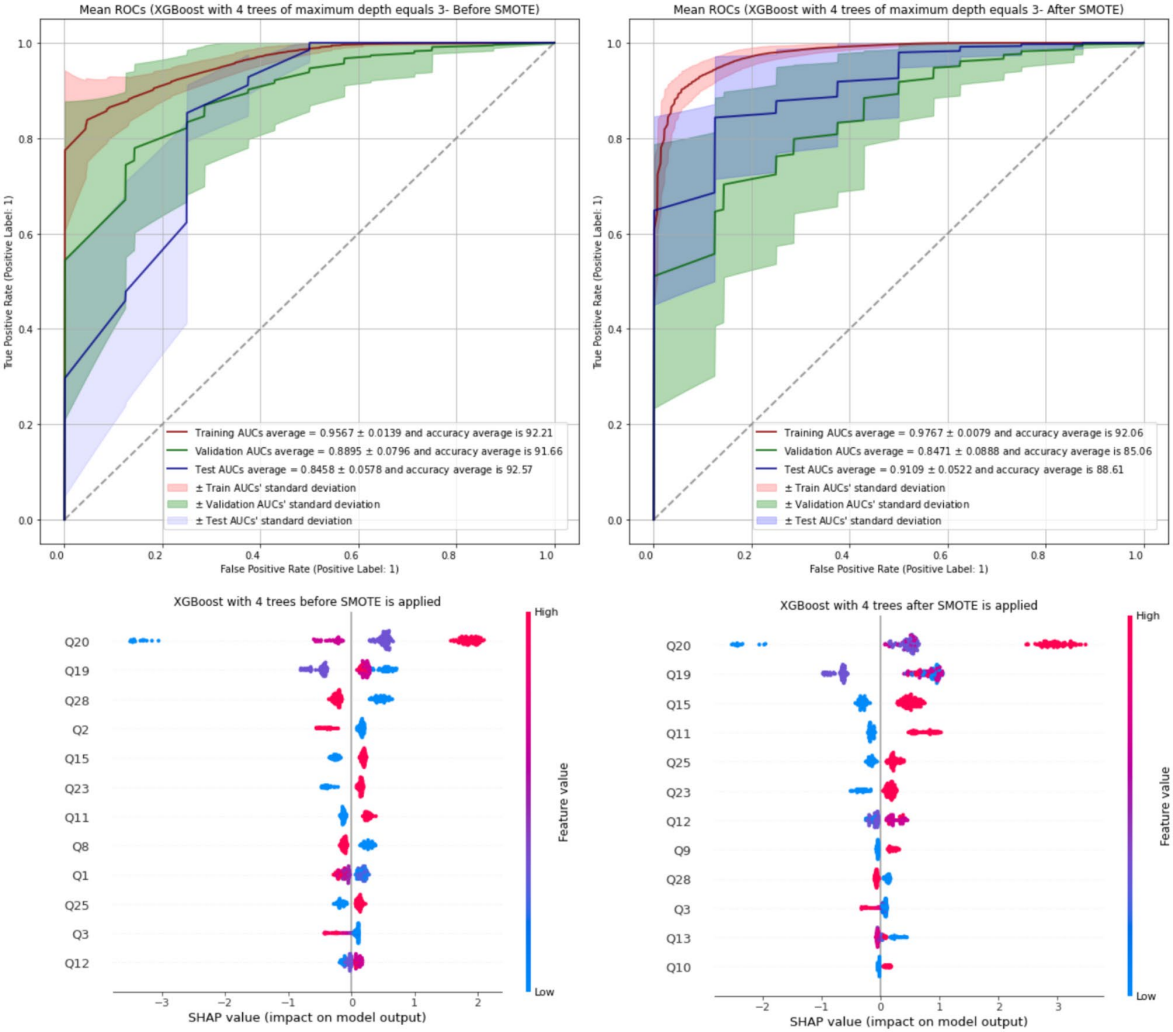
The AUROCs of XGBoost with 4 trees (maximum depth equals 3) on training, validation, and test sets as well as SHAP values of XGBoost with 4 trees of maximum depth of 3 (XGB-4) before and after SMOTE is applied.

XGBoost with 4 trees of maximum depth of tree (XGB-4) has the best performance on averages of validation AUCs and accuracy scores before and after SMOTE is applied. Figure 2 displays the AUROCs of XGB-4 on training, validation, and test sets as well as the top important features of the XGB-4 model by means of SHAP values, which can show how much each predictor contributes, either positively or negatively, to the target variable before and after SMOTE is applied to training sets. Moreover, random forest with 4 trees of maximum depth equals 4 (RF-4), LightGBM with 4 trees of maximum depth equals 4 (LGB-4), and CatBoost with 4 trees of maximum depth equals 4 (CAT-4) have also acceptable performance before SMOTE is applied.

Moreover, Supplementary Material Fig. 3 compares the feature importance scores (percentage) of a random forest containing 4 trees of a maximum depth of 3 (RF-4) with a single decision tree with a maximum depth of 3 (DT-3).

**Figure 3:**
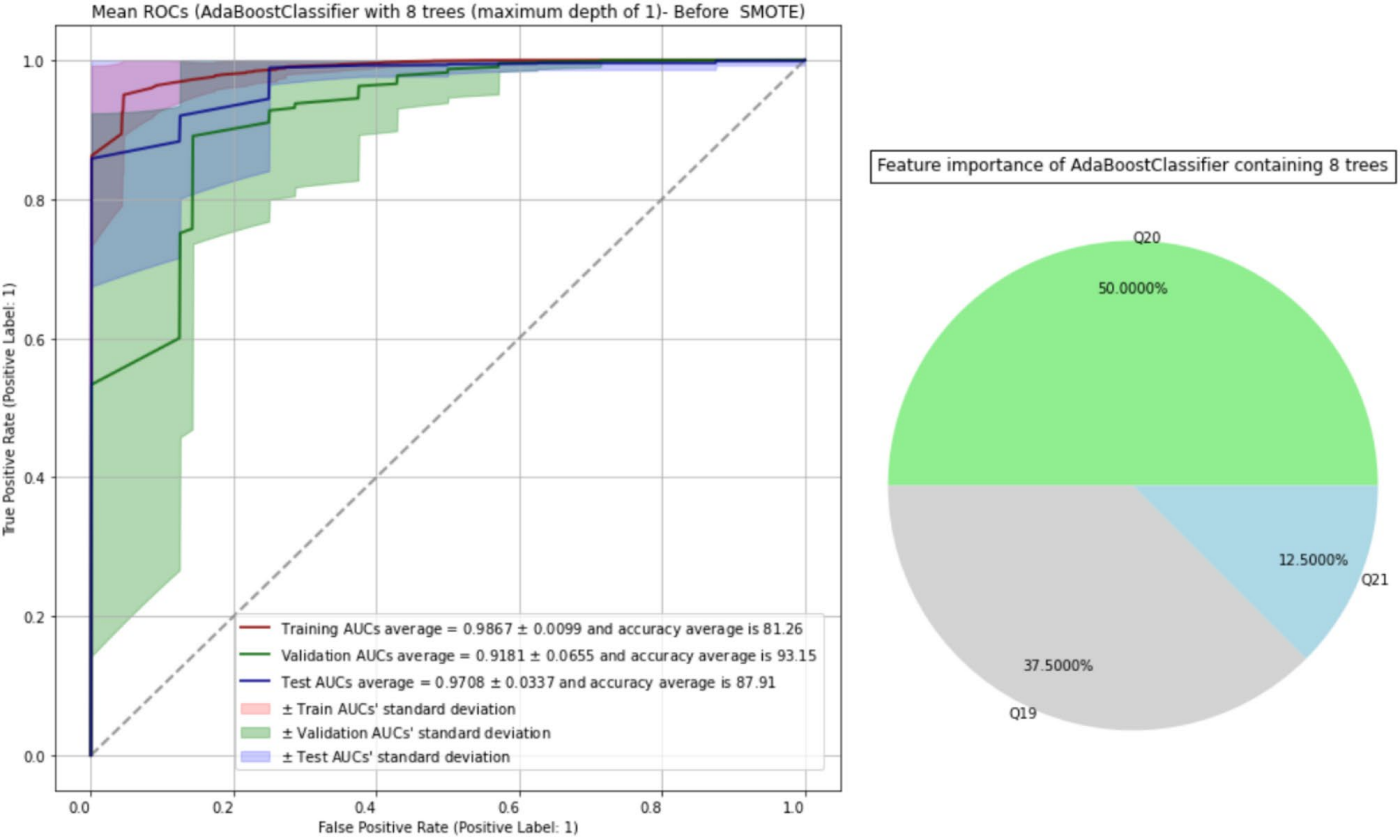
The AUROCs of AdaBoost classifier with 8 stumps on training, validation, and test sets as well as the top predictors of the model.

Table 3 displays the average of AUCs and accuracy scores of AdaBoost with 6, 8, 10 and 15 trees on training, validation, and test sets before and after SMOTE is applied. It turns out that AdaBoost containing 8 stumps (decision tree with depth one) has the best performance on validation sets. Figure 3 displays AUROCs of AdaBoost classifier with 8 stumps (AdaBoost-8) on training, validation, and test sets as well as the top predictors of the model.

**Table 3:** The performance of AdaBoost classifier before and after SMOTE is applied.

Performance (averages)	Model			
	AdaBoost-6 (before SMOTE)	AdaBoost-8 (before SMOTE)	AdaBoost-10 (before SMOTE)	AdaBoost-15 (before SMOTE)
Train AUC	0.9752	0.9867	0.9896	0.9961
Validation AUC	0.8754	**0.9181**	0.8896	0.8739
Test AUC	0.8799	**0.9708**	0.9205	0.9622
Train accuracy	81.07%	81.26%	82.01%	85.32%
Validation Accuracy	92.62%	**93.15%**	92.13%	92.20%
Test accuracy	88.64%	87.91%	92.32%	94.23%

Performance (averages)	Model			
	AdaBoost-6 (after SMOTE)	AdaBoost-8 (after SMOTE)	AdaBoost-10 (after MOTE)	AdaBoost-15 (after MOTE)
Train AUC	0.9779	0.9849	0.9885	0.9941
Validation AUC	0.8803	0.8481	0.8487	0.8472
Test AUC	0.9299	0.8252	0.8376	0.8546
Train accuracy	93.36%	93.57%	94.44%	95.90%
Validation accuracy	88.95%	88.30%	88.03%	89.30%
Test accuracy	88.00%	82.89%	82.64%	84.59%

**Figure 4:**
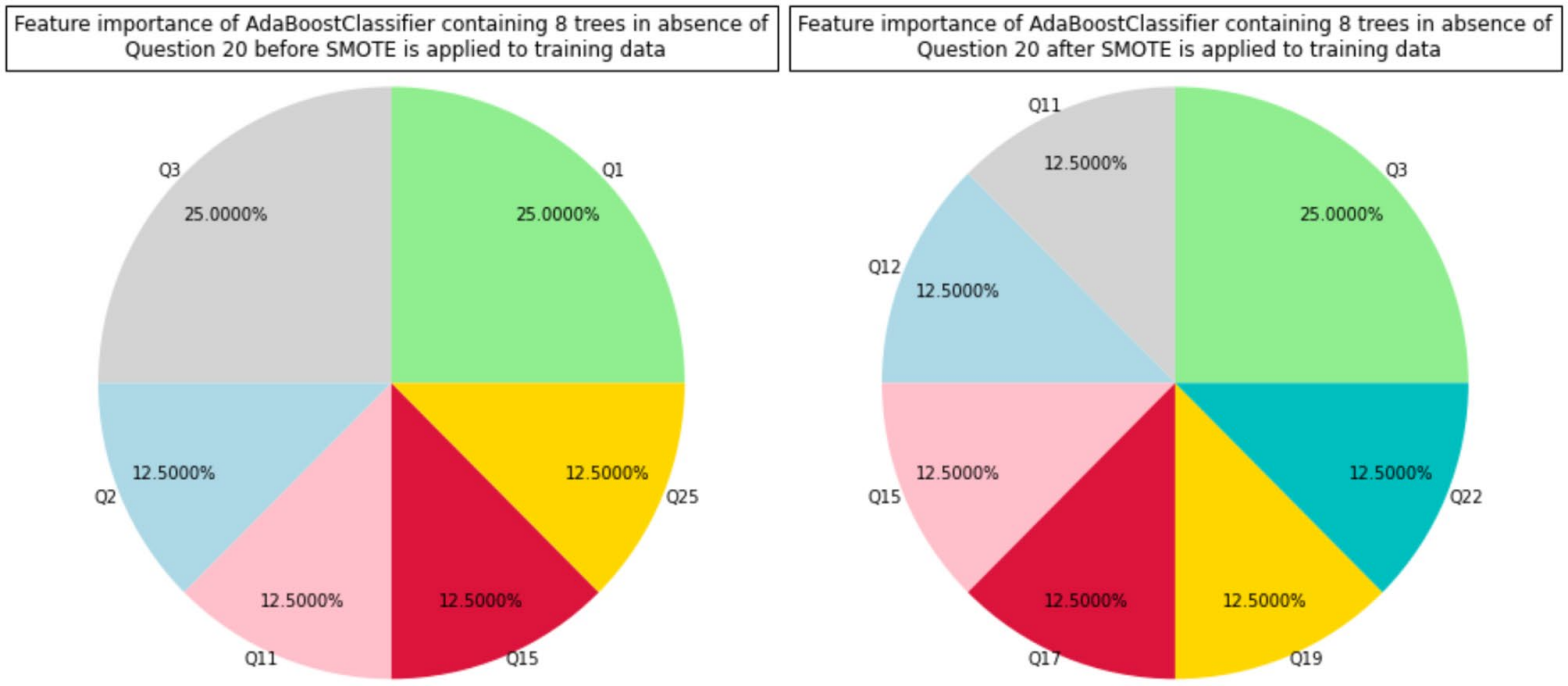
Top predictors of AdaBoost with 8 stumps in absence of Question 20 before and after SMOTE is applied to the training sets.

AdaBoost with 8 stumps is the most accurate and robust supervised model. We remove Question 20 and retrain AdaBoost with 8 stumps. In this way, we can examine other predictors in further details. Without tunning hyperparameters (the number of stumps), we train an AdaBoost with 8 trees on the training sets that were used before removing Question 20 and compute the average of AUCs and accuracy scores on the test sets that were used before Question 20 (see Supplementary Material Section 3). Figure 4 displays the top predictors of AdaBoost with 8 stumps before and after SMOTE is applied to training sets.

### K-Modes clustering

In this section, we first apply K-modes clustering on the data points corresponding to applicants whose response to Question 18a is “I am drinking more alcohol” in an unsupervised manner, and then apply supervised machine learning models within each cluster to find the most important features within each cluster. To determine the number of clusters, we used the elbow method, and it turns out that the optimal number of clusters is three (k = 3). Therefore, we apply K-modes to the group of participants whose response to Question 18a is “I am drinking more alcohol” (86.1% of all data).

To determine the most important common features within each cluster, we create three group (datasets), ${G}_{1}$, ${G}_{2}$, and ${G}_{3}$, using one-vs-all binary cluster labels for all data points. In other words, the new group ${G}_{i}$, for i = 1, 2, and 3, contains all the data points corresponding to applicants whose response to Question 18a is “I am drinking more alcohol,” and each datapoint has a new label: A or B. The label of a datapoint in ${G}_{i}$, for i = 1, 2 and 3, is A if the datapoint belongs to Cluster i, and otherwise B. Note that we use A and B for new groups’ labels to differentiate them from Clusters’ labels, but during the computational process A and B are replaced with 0 and 1, respectively. We then apply tree-based ensemble models, random forest, XGBoost, LightGBM and CatBoost, within each dataset ${G}_{i}$, for i = 1, 2, and 3, to find the most accurate model that can predict the binary label (positive label: A) of the group ${G}_{i}$.

**Figure 5:**
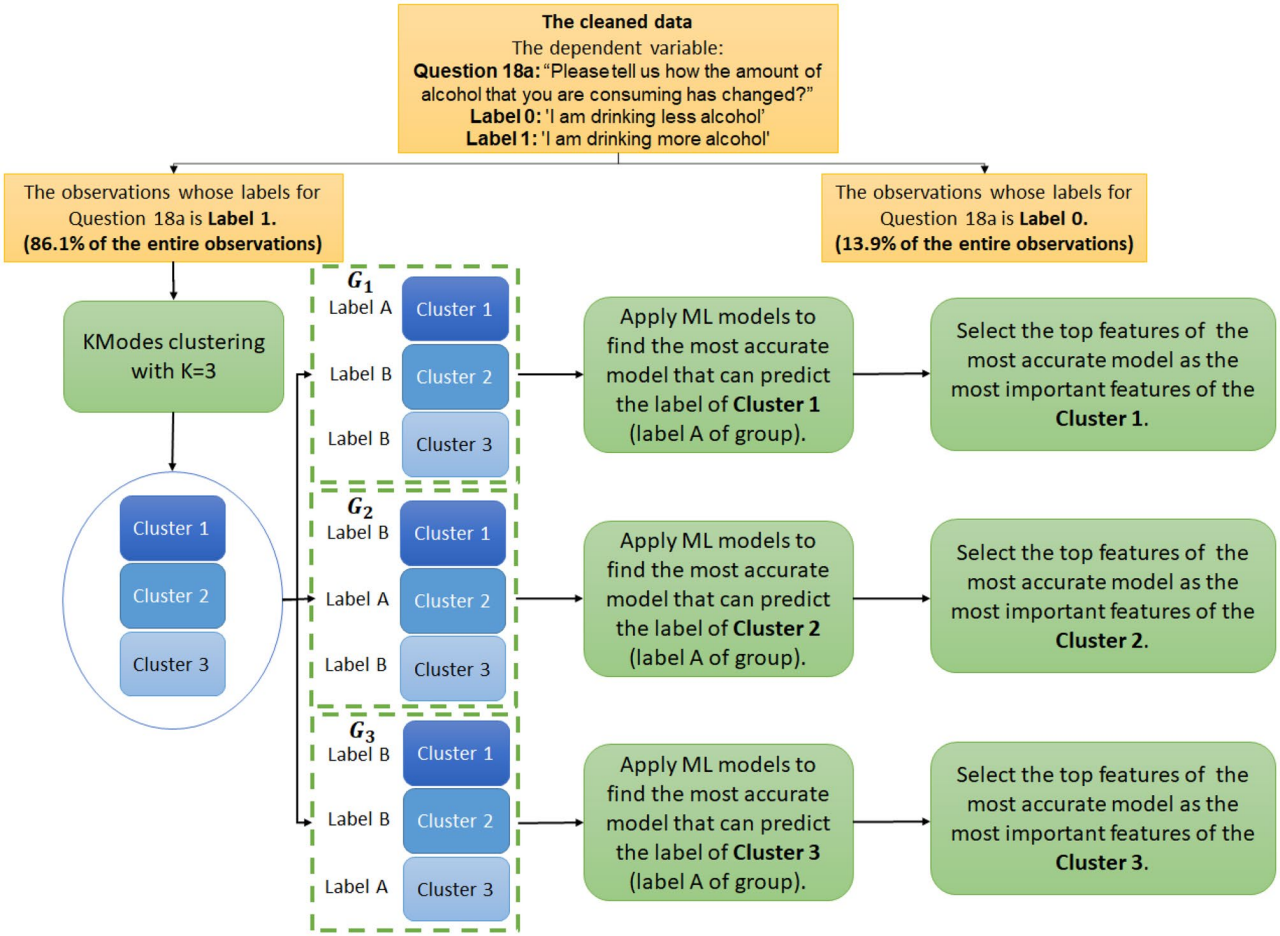
The schematic diagram of clustering method and determining the most important features within each cluster.

Finally, we find the top predictors of the most accurate model within each dataset ${G}_{i}$, for i = 1, 2, and 3, and they are considered as the most important features related to Cluster i, for i = 1, 2, and 3. Figure 5 displays the schematic diagram of the clustering and feature selection process.

**Figure 6:**
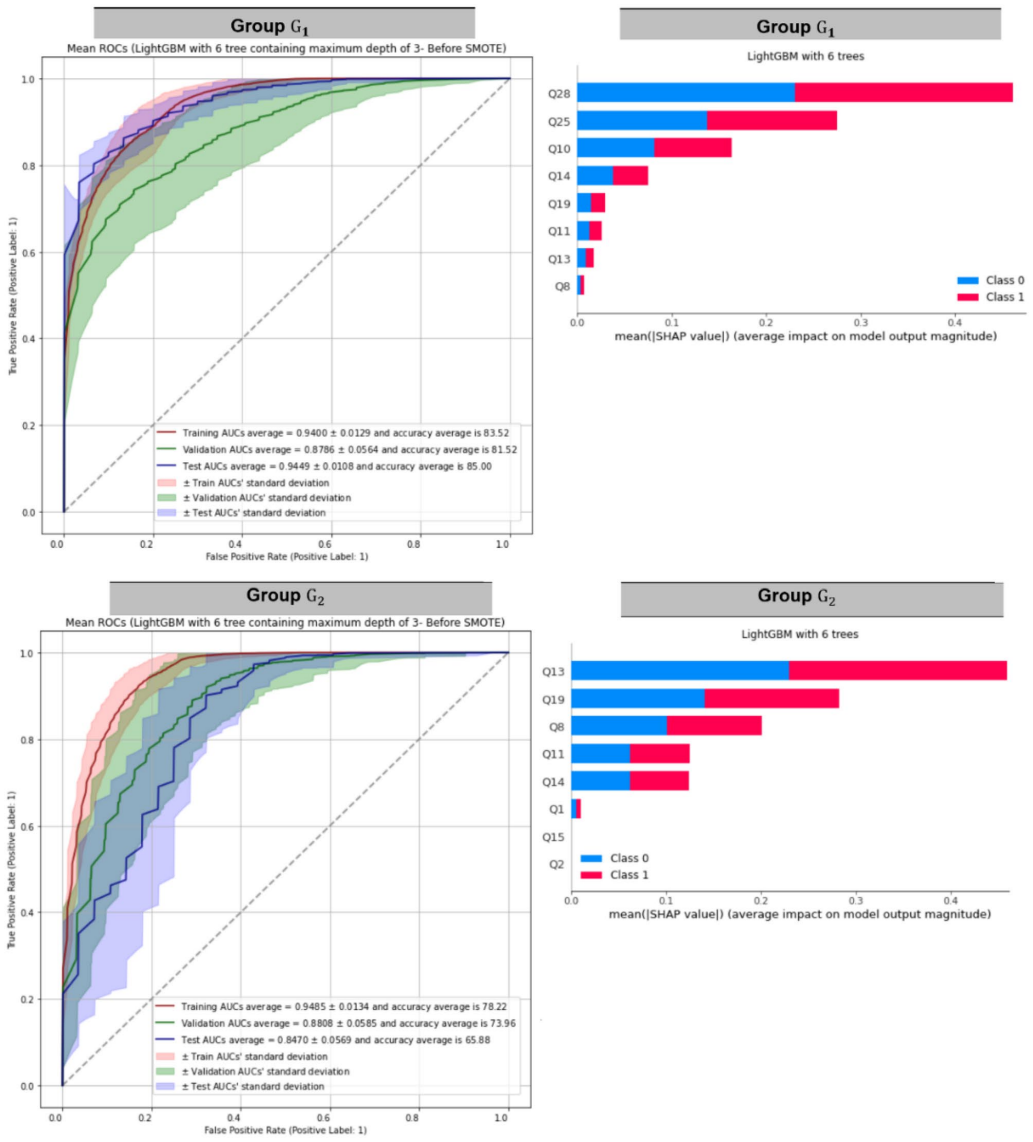

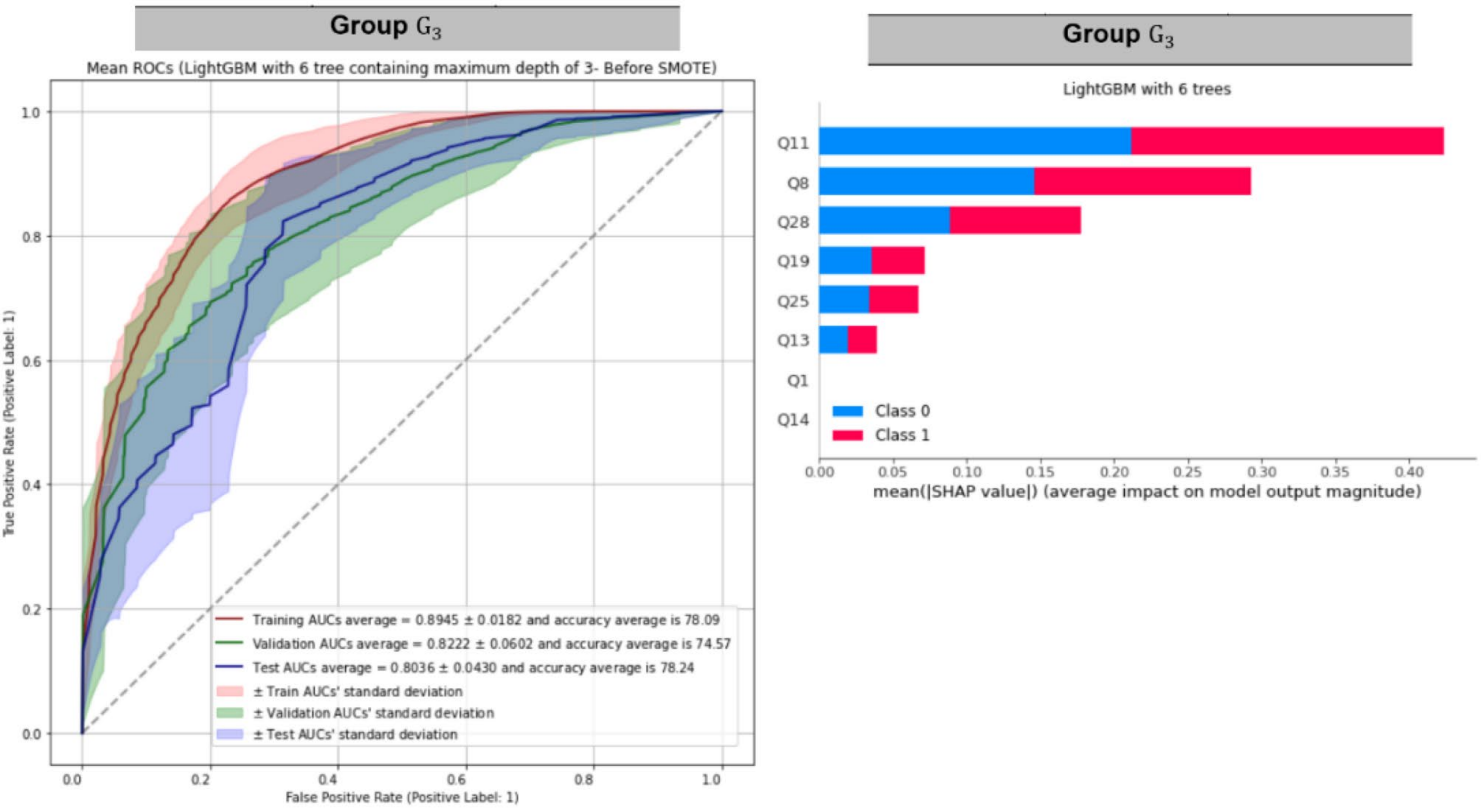
Training, validation, and test AUROCs of LightGBM with 6 trees of maximum depth equals 3 (LGBM-6) as well as the top predictors within each group.

To train models within each group, we use the train-validation-test split stated in Fig. 1. It turns out the models with 6 trees return the best performances. Table 4 displays the performance of each model within each group. Table 4 indicates that LGBM-6 outperforms within each group because its validation and test AUROCs are good, and it does not overfit the training datasets. Figure 6 displays training, validation, and test AUROCs of LGBM-6 as well as the top predictors of LGBM-6 within each group. Figure 6 indicates that the most related features within each cluster are as follow:

- Cluster 1: Q28, Q25, Q10,
- Cluster 2: Q13, Q19, Q8, and
- Cluster 3: Q11, Q8, Q28.

**Table 4:** The performance of random forest containing 6 trees of maximum depth of 3 (RF-6), XGBoost containing 6 trees of maximum depth of 3 (XGB-6), LightGBM containing 6 trees of maximum depth of 3 (LGBM-6), and CatBoost containing 6 trees of maximum depth of 3 (CAT-6) on training, validation, and test sets within each group.

Performance	Model			
	RF-6	XGB-6	LGBM-6	CAT-6
Group ${\mathrm{G}}_{1}$				
Train AUC	0.9409	0.9896	0.9400	0.9898
Validation AUC	0.8559	0.8922	0.8786	0.9364
Test AUC	0.9094	0.9434	0.9449	0.9750
Train accuracy	84.96%	94.28%	83.52%	94.82%
Validation accuracy	77.29%	83.96%	81.52%	87.58%
Test accuracy	79.89%	89.41%	85.00%	90.98%
Group ${\mathrm{G}}_{2}$				
Train AUC	0.9515	0.9885	0.9485	0.9885
Validation AUC	0.8680	0.9235	0.8808	0.9205
Test AUC	0.7892	0.8775	0.8470	0.8674
Train accuracy	85.82%	94.96%	78.22%	94.41%
Validation accuracy	77.47%	83.70%	73.96%	83.59%
Test accuracy	68.83%	78.40%	65.88%	76.68%
Group ${\mathrm{G}}_{3}$				
Train AUC	0.9062	0.9646	0.8945	0.9575
Validation AUC	0.7986	0.8527	0.8222	0.8485
Test AUC	0.8513	0.8831	0.8036	0.8690
Train accuracy	80.99%	89.84%	78.09%	88.59%
Validation accuracy	73.38%	78.70%	74.57%	76.76%
Test accuracy	81.99%	83.48%	78.24%	81.25%

### An overview of top predictors of alcohol consumption habit changes among healthcare workers in the U.S. during the first wave of the Covid-19 pandemic

In this section, we put the top predictors obtained by the accurate supervised, unsupervised, and clustering methods. Note that Question 20: “In the last month, approximately how often did you have a drink containing alcohol?” and Question 19: “In January 2020, approximately how often did you have a drink containing alcohol?” are top predictors for most of the models, but due to the nature of questions, we exclude them from the top predictor list. It turns out that Questions 11 and 15 are the most important features selected by supervised, unsupervised, and clustering methods. Question 11: Both unsupervised methods, Chi-squared test, and mutual information, select Question 11 as the top predictors. XGB-4 before and after SMOTE returns Question 11 as one of the top predictors. The coefficient of Question 11 in logistic regression is a relatively large positive coefficient. Question 11 is one the most important features of RF-4 as well. Question 11 is also the most important feature within Cluster 3 when KModes clustering is applied to the data points corresponding to applicants whose response to Question 18a is “I am drinking more alcohol.” Finally, Question 11 is one of the top predictors of the AdaBoost-8 classifier before and after SMOTE is applied to the training sets.

Question 15: Both unsupervised methods, Chi-squared test, and mutual information select Question 15 as the top predictors. Coefficient of Question 15 in logistic regression is the largest positive coefficient. XGB-4 before and after SMOTE returns Question 15 as one of the top predictors. Question 15 is also one of the most important features of RF-4. Finally, Question 15 is one of the top predictors of AdaBoost-8 classifier before SMOTE is applied to the training sets. After SMOTE Question 15 is the most important feature of AdaBoost-8. Question 28: This question is the top predictors of XGB-8 before SMOTE and one the most important features after SMOTE. Moreover, Question 28 is selected as the most important common feature in Cluster 1. Table 5 displays some of top predictors that selected by the best supervised method (AdaBoost-8, XGB-4), unsupervised methods (Chi-squared, mutual information), and clustering (by LGBM-6).

**Table 5:** Top predictors selected by supervised, unsupervised and clustering methods.

Top predictors	Methods
Q11: Are children home from school in the house? Encoded responses: No: 1; Yes: 2	Chi-squared, mutual-information, XGB-4, RF-4, Clustering, logistic regression, AdaBoost-8
Q15: Have you varied your work schedule? Encoded responses: No: 1; Yes: 2	Chi-squared, mutual-information, XGB-4, RF-4, logistic regression, AdaBoost-8
Q28: Has the amount of food you have been eating per day changed? Encoded responses: No: 1; Yes: 2	Clustering, AdaBoost-8
Q1: What is your age? Encoded responses: In 20 s: 2; 30 s: 3; …; 80 s:8	AdaBoost-8, XGB-4
Q2: What is your gender? Encoded responses: Female: 1; Male: 2	AdaBoost-8, XGB-4
Q3: Where do you live? (50 States, D.C. and Puerto Rico)	AdaBoost-8, XGB-4
Q12: Approximately how many hours did you sleep on an average work night in January 2020? Encoded responses: Between 2–10	AdaBoost-8, XGB-4
Q13: Approximately how many hours did you sleep on an average work night in the last week? Encoded responses: between 3–10	Clustering, XGB-4
Q23: Has the amount of news you are consuming increased since the end of Februrary, 2020? Encoded responses: No: 1; Yes: 2	XGB-6, logistic regression
Q25: Have you had more “screen time” (e.g. use of smartphone, tablet, etc.) around bedtime? Encoded responses: No: 1; Yes: 2	XGB-4, Clustering, logistic regression, AdaBoost-8
Q8: Are you currently conducting your job mostly from home now? Encoded responses: No: 1; Yes: 2	Clustering, XGB-4

### Comparison study

In this section, we will examine our findings in relation to other studies that have been conducted in a similar area. It is important to highlight that our study is unique in that it incorporates advanced supervised and unsupervised methods that have not been utilized in previous research. Table 6 presents a comparative analysis of our research approach, outcomes, and the survey location with that of other studies conducted in different geographic locations. Note that the study by Beiter et al.^12^ reported that alcohol consumption was associated with being White. However, since a large majority of the study's participants self-identified as White (n = 80, 78.4%), it is unlikely that being White is a useful predictor.

**Table 6:** A comparison of our study methodology and findings with those of similar studies conducted in various geographic locations.

Studies	Methods	The geographical location where the survey was conducted	The most associated predictors of higher alcohol consumption
Beiter et al.^12^	Alcohol Use Disorders Identification Test–Consumption (AUDIT–C)	Louisiana, US	White race, using avoidant coping, being single, having depression symptoms, and having a strong emotional reaction to the pandemic
Mongeau-Pérusse et al.^13^	Wilcoxon signed rank test for categorial variables and paired-t tests for continuous variables	Quebec, Canada	Prolonged social isolation, psychosocial stressors, and the use of alcohol as a self-medication for anxiety, stress, and worry related to the pandemic
Pomazal et al.^14^	Chi-squared tests, and logistic regression	Wisconsin, US	Younger age, anxiety, higher education levels, higher income, working remotely, and having children at home
**Our study**	Decision trees, logistic regression, support vector machines, multilayer perceptron, XGBoost, CatBoost, LightGBM, AdaBoost, Chi-Squared Test, mutual information, KModes clustering and the synthetic minority oversampling technique (SMOTE)	Michigan, US	Having children at home, A change in work schedule, changes in food consumption, age, gender, geographical characteristics, changes in sleep habits, the amount of news consumption, social media exposure and screen time

## Discussion

In the previous section, we examined some unsupervised and supervised machine learning methods to determine the top predictors of alcohol consumption habit changes among healthcare workers in the United States. Some of the questions from the survey (predictors) have been selected by the most accurate supervised models. Chi-squared test, mutual information support, and K-modes clustering support some results obtained by supervised methods. In this section, we discuss the top predictors, and we explore some non-machine-learning based articles whose results agree with our analysis.

One of the most important questions, or features, that appears in all unsupervised learning methods and supervised learning algorithms is Question 11 that reads, “Are children home from school in the house?” It raises a question about the relationship between COVID-19-associated school closure and alcohol consumption habit changes among healthcare workers. There is a strong relationship between COVID-related school closure, parenting stress, and alcohol consumption that requires further research of the topic. Based on our findings, the school closure associated with the COVID-19 pandemic can be associated with an increase in alcohol consumption as a coping mechanism. To alleviate the hidden effects of COVID-19, e.g., parenting stress, on healthcare workers, some operational strategies might be used in schools to reduce the spread of COVID-19 and maintain safe operations in school during the COVID-19 pandemic. Boschuetz et al.^58^ discuss how alcohol use patterns differ from the effects of social distancing caused by Covid-19. They use the Alcohol Use Disorders Identification Test (AUDIT-C) to measure alcohol use. A higher score on the test indicates higher levels of drinking in patients and that drinking could lead to harm within the patient.

A Chi-squared test was used to compare the original results from before social distancing to results obtained after social distancing was implemented. Additional tests such as the paired t-test and McNemar test were used to compare the different variables. They conclude that having children at home was deemed statistically significant as values for the AUDIT-C test were on average 0.47 for having children at home and 0.1 for not having children at home. The p-value for having children at home was 0.01, deeming the presence of children at home statistically significant. In a multivariable analysis, the presence of children at home remained statistically significant.

Another interesting point brought forth by this paper was the difference in AUDIT-C scores between men and women. The researchers in this study believe the difference in values of women and men with children at home could be due to the notion that women are usually responsible for mainly raising the children. Homeschooling the children could also be a factor increasing the stress in women over men as the study notes that oftentimes women are tasked to homeschool their children while working from home. The difference between males and females had a p value of 0.03, making the data statistically significant. This could be why in the study Question 11 is one of the most influential questions in determining alcohol consumption. Acuff et al.^59^ compare the results of various empirical studies conducted between the Summer and Fall of 2020 that dealt with drinking practices at the start of the pandemic. A subset of the studies considered other factors such as sex, age, and children at home in determining the results of the study. The number of articles included in the final meta-analysis was 128 papers with data on the drinking effects that were caused by the pandemic. The study concluded that each additional person that was in a household was associated with a greater increase in drinking. Parents living with children displayed a higher increase in drinking as well, which displays how living with more people in the house can lead to higher levels of stress.

Both the Chi-Squared test and Mutual-Information method, as well as some supervised learning algorithms imply that Question 15: “Have you varied your work schedule?” is not independent of alcohol consumption habit changes among healthcare workers in the US. Our results support a study by the Ohio State University College of Nursing^60^, which reveals that nurses working long shifts due to the COVID-19 pandemic have experienced negative effects. In another study by Cooper et al.^61^, it is shown that work stressors lead to increased distress, which in turn promotes problematic alcohol use. They also reveal the relationship between work-related stress and an increase in alcohol consumption among healthcare professionals during the COVID-19 pandemic.

Ahn^62^ examines the relationship between work hours and drinking habits and concludes shortening work hours increases the probability of participating in drinking but does not significantly increase daily drinking habits. Frone^63^ investigates a pattern between job support and alcohol consumption. From the Cause-and-Effect Model, they have suggested that jobs low in complexity and control and high in demand are related to increased employee alcohol use. However, the results from the Cause-and-Effect Model are inconsistent, and that is why they used the Moderation Model. Then, they found a positive association between job demands and lack of a clearly defined role at the workplace (role ambiguity) and heavy drinking.

As the COVID-19 pandemic continues, healthcare workers are more in need of working shifts, and consequently, healthcare workers who work shifts may consume more alcohol than day workers. Therefore, using results from^63^ and our ML-based results, we can conclude that work stress related to Covid-19 and a change in work schedule is positively associated with job dissatisfaction, which causes an increase in alcohol use among vulnerable people.

Question 28: “Has the amount of food you have been eating per day changed?” is selected by AdaBoost-8 and K-modes clustering as one the most important top predictors. This supports the findings in^64^ and^65^. Huber et al.^64^ surveyed 1980 students about their eating habits during Covid-19. Questions were asked about the setting where participants eat their food, the amount of food consumed, and the healthiness of the food. Demographic information such as age, height, and gender was recorded in the survey.

In the second part of the study, participants were asked about their food consumption along with smoking and alcohol consumption since the start of the pandemic. SPSS 25 was used to perform a statistical analysis of the data. Categorical variables were compared using Chi-squared tests to determine a relationship between data points, and non-categorical variables were compared using Kruskal–Wallis tests. Their results concluded that people’s eating habits had drastically changed during the pandemic. People were eating an increased number of homecooked meals and were eating at restaurants very rarely. Almost 50 percent of people reported eating in restaurants or cafeterias before the virus, but now less than 3% of people reported eating in these places. Half of the participants stated that the amount of food consumed had not changed during the lockdown, while 31% said their food intake had increased and 16% stated that their food intake had decreased. Using a bivariate analysis, the study concluded that alcohol consumption showed a significant correlation with the change in food consumed. The study concluded that changes in alcohol drinking patterns led to an increase in food intake in their sample population.

Yeomans et al.^65^ discuss the relationship between alcohol use and food consumption in individuals. They noted that previous findings also determined that alcohol consumption prior to a meal leads to a short-term increase in food intake. They found that the short-term effects of alcohol have both direct and indirect effects on the amount of food consumed. According to their findings, food intake is increased in the period short after consuming alcohol due to the pharmacological and energetic effects alcohol induces on the body.

Question 1: “What is your age?” is selected as an important feature by AdaBoost-8 and XGBoost-4. Villanueva-Blasco et al.^66^ showed that as age increases, the percentage of consumers of alcohol who increased their consumption during the pandemic is higher. In daily alcohol consumption the lower age ranges had a higher decrease in daily alcohol consumption than the older age ranges. They concluded that age is a large factor in alcohol consumption during the pandemic. The limitations of alcohol consumption by younger adults can be explained by the closure of venues due to the pandemic. There are less social drinking opportunities, and therefore younger adults have less reason to drink.

For older adults, alcohol consumption is more associated with their home and, therefore could be a reason for the increase in consumption. Härkönen et al.^67^ utilized six Finnish Drinking Habits Surveys, which sampled a population between the ages of 15–69. All the surveys were conducted as interviews and asked questions to measure the frequency of light and binge drinking occasions. Then they used a binomial linear regression to model the distribution after the number of light and binge drinking occasions per year. They concluded that light drinking increased between the ages of 15–29 in both women and men. After the age of 29, light drinking in men continued to increase until about age 54 before plateauing until age 69. However, in women, light drinking seemed to have a slight decrease after the age of 29. Similar to light drinking, binge drinking increased within the male and female cohorts until around age 37. In males, binge drinking habits continued to increase until around age 61, while in females, these habits decreased after the age of 37. This will provide insight into why age is a pivotal factor in determining the amount of alcohol consumption, especially during the Covid-19 pandemic. Therefore, regarding the relationship between age and alcohol consumption changes, we may utilize our findings, which agree with the findings of^68^, to make a conclusion: the susceptibility to stress differs between young and old people, and may lead to different stress responses e.g., turning to alcohol to cope with stressful situations.

Question 2: “What is your gender?” is selected as an important feature by AdaBoost-8 and XGB-4. Villanueva-Blasco et al.^69^ discussed the patterns of alcohol consumption during the Covid-19 pandemic based upon gender in Spain. Their study used convivence sampling through online surveys that surveyed a group of 3779 participants. 70% of the participants were female and 30% of the participants were male that were surveyed. The average age of all participants was 37.76 years old.

Data collection began after confinement and continued until measures eased up. AUDIT-C was used to measure alcohol consumption in the study. Participants were asked about their drinking habits 6 months into confinement compared to their drinking habits before the onset of the pandemic. Different data analysis strategies were employed, such as the Chi-squared test and frequency analysis. Their results indicated that in both males in females, there was an increase in daily average consumption of 1–2 drinks per day but a decrease in daily consumption of 3–4 drinks per day. However, for consuming alcohol more than 4 days a week, the number of females during confinement doubled, whereas for males the number only increased by a factor of 1.5. For both males and females, the frequency and average daily consumption decreased at a similar rate; however, for females, the frequency of intensive consumption and the average SDUs or drinks per day decreased at a lower rate than that of males. This leads to a possible correlation between gender and alcohol consumption during the pandemic.

The researchers possibly attribute this discrepancy between gender to the fact that females drink more within the home and males in more social settings. This would lead to an increase in alcohol consumption in females compared to males. The main subgroup affected by the pandemic was heavy drinkers as both males and females started to consume 4 or more drinks per week. This can be attributed to buying alcohol as a weekly purchase and having the pleasure of drinking at home because most people had to work at home during this time. So, regarding the relationship between gender differences and alcohol consumption changes during the first wave of Covid-19 pandemic, we may make a conclusion, which agrees with the results of^70^: men and women tend to react differently with stress, both psychologically and biologically, and may lead to different stress responses.

Question 3, which is another important predictor, asks where the healthcare workers live (50 States, D.C., or Puerto Rico). Klein et al.^71^ describe the regional differences in alcohol consumption. The study attempts to determine whether the region of residence influences alcohol consumption. Results indicated that geographic region has little determining effect on people’s alcohol use. However, the study did find a small impact between geographic region and drinking-related attitudes. Brenner et al.^72^ found that there exists a considerable regional variation in the amount of alcohol consumed. Their results showed that there was a significant state of residence impact on alcohol use. They also found a correlation between rural versus urban residence. They concluded that living in a small town compared to a metropolitan area was associated with less frequent drinking. In addition, living in a rural area compared to an urban area was associated with more frequent alcohol consumption. Our results, besides results in^71^ and^72^, can justify the importance of where a healthcare worker lives on their alcohol use habit changes. During the first wave of Covid-19, most of the hospitals in populous states were mostly occupied by Covid-related patients. Hence, the healthcare workers in populous states experienced more work-related stresses which were associated with an increase in alcohol use consequences for some healthcare workers.

Question 12: “approximately how many hours did you sleep on an average work night in January 2020?” is selected as an important predictor. Imaki et al.^73^ analyzed the relationship between the hours of sleep and lifestyle factors in Japanese factory workers. In this study, they compared lifestyle factors for factory workers who got 6 or fewer hours of sleep, 6.1–8.9 h of sleep, and 9 or more hours of sleep, and they concluded that there is no significant difference between alcohol drinking between the groups who got 6 or fewer hours of sleep and 6.1–8.9 h of sleep. The study notes, however, that the group receiving more than 9 h of sleep was excluded from the analysis because they only made up 1% of the participants. Miller et al.^74^ conducted a study that examined if adequate sleep is associated with drinking quantity and its consequences. In their study, College students reported drinks consumed per week, average sleep quality, and sleep adequacy. They found significant interactions between adequate sleep and weekly drinking quantity. In addition, this interaction was associated with alcohol-related consequences. Based on our ML results, as well as results in^73^ and^74^, we may conclude that the Covid-related issues caused an increase in alcohol use, and it subsequently led to changes in sleep patterns in healthcare workers.

Question 13: “How many hours do you sleep on an average night?” is another predictor that is selected as an important predictor. In their study, Du et al.^75^ sampled students from various countries who were over the age of 18. The studies were conducted during times when the countries were under strict shelter-in-place orders. Demographic data such as age, sex, class status, height, and weight were all recorded in the studies. Perceived stress, alcohol consumption, and sleep duration were all recorded through questionnaires of the various students to determine relationships between these variables and the ongoing Covid-19 pandemic. Alcohol misuse was measured using the AUDIT-C questionnaire, and sleep was assessed using the Pittsburgh Sleep Quality Index. Questions were asked about how the Covid-19 pandemic affected various factors in their life including sleep quality and alcohol consumption. IBM SPSS version 26 was used to perform descriptive statistics, and statistical significance tests.

A total of 28 comparisons were used between the data in order to determine relationships between sleep quality and alcohol consumption during the pandemic. A total of 2254 students were analyzed for this study. 67% of the students were female and 58.7% of the students were living in the United States. Their results concluded that alcohol use was positively correlated with greater perceived stress and poor sleep quality. Poorer sleep quality meant a shorter duration of sleep which was increased with alcohol use. Improving sleep quality in these students would lead to a decrease in alcohol use which could explain why the question of how many hours one sleeps per night affected alcohol consumption. A decrease in sleep quality also leads to a positive correlation with stress, and the association between stress and alcohol misuse was statistically significant which could also impact the use of alcohol during the pandemic.

Question 23: “Has the amount of news you are consuming increased since the end of Feb, 2020?” is another important predictor that is selected by XGB-4. Chartier et al.^76^ investigated the relationship between social media use and alcohol consumption during the first onset of the Covid-19 pandemic. Their data was taken from the Understanding American Study which is a group of 5,874 individuals who are 18 years or older that are meant to resemble the United States Population. The participants completed an original survey in March of 2020, and a follow-up survey one month later. The data were analyzed to determine any relationships between social media use and alcohol use frequency. Their analysis compared the social media use of participants in March 2020 with their alcohol use a month after. Hierarchical logistic regression was employed to examine how increased and decreased alcohol use altered with social media consumption. Their results concluded that social media use did not affect drinking habits during March 2020; however, alcohol use was higher for Wave 3 in individuals who had higher levels of social media use.

Alcohol use was also higher for individuals who used multiple social media platforms than those who only had a single social media account. They believe that individuals who had higher amount of social media activity would be exposed to more news content. This content especially surrounding the virus had been previously shown to lead to higher levels of stress in individuals which could account for the increase in alcohol use. Also, during the pandemic, many workers were forced to work from home and had more free time to scroll social media which could have sparked a major increase in social media use. This increase in social media activity would lead to greater consumption of news which could account for the reason so many individuals reported an increase in news consumption. Engels et al.^77^ found that the portrayal of alcohol in the movie and commercials directly affected alcohol consumption during the movie.

On average, people who were presented with alcohol portrayal in the movies and in the commercials consumed around 1.5 more drinks than those presented with no alcohol portrayal. This could provide insight into how consuming the news affects alcohol use because when watching the news, viewers would be susceptible to alcohol advertising which could lead to increased use. Strainback et al.^78^ also explore COVID-related news effects on mental health. Their findings show that COVID-19 media consumption leads to psychological distress, which may cause an increase in alcohol consumption. Thus, we can make a conjecture: the stress resulting from COVID-19 related news exposure may produce changes in drinking behavior. Question 28: “Has the amount of food you have been eating per day changed?” is another important feature that is selected by LightGBM and CatBoost. An increase in alcohol consumption can cause changes in a healthcare worker's diet. People who drink alcohol more frequently may choose less healthy food options that are high in fat and sugar^79^. Moreover, each gram of pure alcohol has 29 kilojoules of energy^80^, and when it is mixed with sugary drinks, it contains even more calories. Since alcohol is absorbed directly in the bloodstream^81^ that can lead to immediate changes in the amount of food that people eat.

Question 25: “Have you had more screen time around Bedtime?” is also selected by AdaBoost-8 and XGB-4. Tebar et al.^82^ investigated how the increase in screen time that was caused by Covid-19 isolation policies influences the consumption and desire for alcohol in the U.S. population. In their study, a survey was conducted on 1897 adults with a mean age of 38. Participants were asked questions about their screen time habits during the Covid-19 pandemic, along with questions detailing their alcohol consumption and smoking habits. Demographic information detailing their gender, age, race, and working conditions were recorded in the survey as well. Overall, the survey was comprised of 70 questions to assess the various levels of screen time on different devices and their addictive behaviors. Binary logistic regression models were employed to discover the relationship between an increase in screen time and various addictive behaviors.

Their results showed that individuals who reported an increase in television time had an increased desire to drink during the pandemic. However, individuals who reported that computer time increased during the pandemic were less likely to report alcohol consumption. Increased cell phone time was associated with an increase in alcohol consumption during the pandemic as well. The study concluded that increases in cell phone and television consumption were factors influencing the increase in alcohol consumption and desire to consume alcohol. The study attributes these increases mainly to advertisements that contain alcohol being displayed on both television and cell phone. Overall, screen time was concluded to lead to an increase in addictive behaviors such as smoking and drinking.

Question 8: “Are you currently conducting your job mostly from home now?” is another important predictor. This correlates to having children at home because in^58^ they found that when working from home parents were often tasked with raising and teaching their kids during the pandemic which leads to extra stress. Schmits et al.^83^ used a sample of 2871 adults were asked to report on an online questionnaire. Participants were between the ages 18 and 85 and mainly lived in France and Belgium. As of the Covid Lockdown 36.5% of participants in the study were working from home. Demographic data was captured along with their living environment and how it changed during the lockdown. The frequency and quantity of alcohol use of the respondents was surveyed and assessed through an adapted version of the AUDIT-C questionnaire. An additional item was incorporated into the studies to evaluate how their alcohol use changed from before the lockdown. They were asked a variety of questions about who they consume alcohol with, how much alcohol they consume, and what factors lead to them consuming alcohol. Then, they used SPSS 26 software to perform various statistical tests such as Chi-squared, descriptive statistics, Kruskal–Wallis, and Spearman’s correlation tests. They finally concluded that individuals that were working from home tended to increase their alcohol use during lockdown by an average of 36.4%. The research also concluded that patients who were busier during lockdown increased their alcohol intake as well. The researchers attribute this increase in alcohol consumption at home to the absence of workplace regulations and the availability of the substance at home.

Children could also lead to increased stress among parents, where working at home would lead to increased time under stress from their children. Finally, the researchers hypothesize that the consumption increase could be in response to shifting from a work environment to a home environment. In a work environment, alcohol is less regulated, and therefore when one is working from home, they are around a higher-risk environment. In another research by Caluzzi et al.^84^, it was found that people who lost their jobs or switched to working from home had increased their alcohol consumption. Their findings suggest that the normal obligations and restrictions that would limit one’s drinking had been reduced by working from home and their daily routine had been completely changed. This quick change can lead to increased stress and allows individuals to lose accountability for their actions. The subjects felt that while working from home the weekends and weekdays were blurred together, which could often lead to drinking habits during the week instead of only on the weekends. These are all reasons why working from home has increased the amount of alcohol consumed.

Our study revealed that healthcare workers who had children at home during the first wave of Covid-19 in the US had increased alcohol consumption. Moreover, we observed that changes in work schedules caused by the pandemic were associated with changes in alcohol use habits. Other factors that were found to be significant predictors of increased alcohol use among healthcare workers in the US included changes in food consumption, age, gender, geographical characteristics, changes in sleep habits, the amount of news consumption, and screen time. Table 6 provides a comparison between our research methodology, results, and the location where the survey was conducted with those of other studies carried out in various geographical regions. It should be noted that similar top predictors for increased alcohol consumption were identified in both our study and the study conducted by Pomazal et al.^14^. It is worth mentioning that while our analysis was based on a survey conducted in Michigan, the study by Pomazal et al.^14^ was conducted in Wisconsin. Although our study identified children at home as the top predictor of alcohol consumption increase during the pandemic, the study by Pomazal et al.^14^ also identified it as an important factor. Moreover, both our study and the study conducted by Pomazal et al.^14^ identified age as a top predictor of increased alcohol consumption during the COVID-19 pandemic.

## Conclusions

This paper focused on the impact of the Covid-19 pandemic on alcohol consumption habits among healthcare workers in the United States during the first wave of the pandemic. To investigate the links between COVID-19-related deleterious effects and changes in alcohol consumption habits, we utilized multiple supervised and unsupervised machine learning methods and models such as such as decision tree, logistic regression, k-nearest neighbors, support vector machines, multilayer perceptron, XGBoost, CatBoost, LightGBM, AdaBoost, Chi-squared test, mutual information method, and K-modes clustering on a mental health survey data obtained from the University of Michigan Inter-University Consortium for Political and Social Research. Through the interpretation of several robust and accurate supervised models as well as unsupervised methods applied to the dataset, we found that healthcare workers whose children stayed home during the first wave in the US consumed more alcohol. We also found that the work schedule changes due to the Covid-19 pandemic led to a change in alcohol use habits. Changes in food consumption, age, gender, geographical characteristics, changes in sleep habits, the amount of news consumption, social media exposure and screen time are also important predictors of an increase in alcohol use among healthcare workers in the United States. In future work, we are interested in analyzing more data related to healthcare workers to examine the relationship between their mental health and other factors related or unrelated to COVID-19. As new viruses continue to emerge, it is important that we learn from past experiences and prepare for any possible pandemics. This includes taking steps to ensure the safety and well-being of healthcare workers.

## Supplementary Information

Supplementary Information.

## Data Availability

The data that support the findings of this study are available from the University of Michigan’s Inter-University Consortium for Political and Social Research, which is collected by Deirdre Conroy^37,38^, at https://www.openicpsr.org/openicpsr/project/127081/version/V1/view?path=/openicpsr/127081/fcr:versions/V1&type=project.
