# Supplementary material for: Machine learning-based analytics of the impact of the Covid-19 pandemic on alcohol consumption habit changes among United States healthcare workers
Source: Sci Rep. 2023 Apr 12;13:6003. doi: 10.1038/s41598-023-33222-y (PMC10092930; doi:10.1038/s41598-023-33222-y)
Supplement: Supplementary file 1 — Supplementary Information. [file 41598_2023_33222_MOESM1_ESM.docx]

**Supplementary Material for**

**Machine Learning-based Analytics of the Impact of the Covid-19 Pandemic on Alcohol Consumption Habit Changes Among United States Healthcare Workers**

**Mostafa Rezapour^1*^, Muhammad Khalid Khan Niazi^1^, Metin Nafi Gurcan^1^**

^1^Center for Biomedical Informatics, Wake Forest University School of Medicine, Winston-Salem, NC, USA

^*^Corresponding author: [mrezapou@wakehealth.edu](mailto:mrezapou@wakehealth.edu)

1. **Data preprocessing**

The COVID Isolation on Sleep and Health in Healthcare Workers data is a tabular dataset with 916 rows (data points or participants) and 64 columns (features or attributes) such as StartDate, EndDate, IPAddress, etc [1]. The dataset contains 14678 missing values, with 480 missing values for Question 28a, 311 missing values for Question 29a, and so on. This paper focuses on Question 18a: “Please tell us how the amount of alcohol that you are consuming has changed?” as the target variable.

To prepare the dataset for a machine learning analysis with Question 18a as the target variable, we first remove all the rows with no value (answer) in the column corresponding to Question 18a. We then remove unrelated columns such as StartDate, EndDate, Status, etc., reducing the number of missing values to 1612. Most of the variables in the dataset are categorical (nominal or ordinal), but since this is a COVID-related analysis, we would prefer not to use machine learning techniques or models (e.g., KNN) to treat the missing values for the categorical variables. Instead, we directly remove the rows with too many missing values from the dataset, and as a result, we end up with 273 clean rows (with no missing value). Since the mental health dataset contains categorical and ordinal variables, we first encode them into numbers. We then use several encoding techniques, such as one-hot-encoding or dummy variable encoding as well as several packages in Python, such as OneHotEncoder, LabelEncoder or OrdinalEncoder from sklearn preprocessing to prepare the dataset for analysis. **Table 1** displays the distribution (the relative frequency) of data points of the cleaned data between two classes of Question 18a.

Table 1. Distribution of responses between two classes of the dependent variable.

| **Question 18a:** “Please tell us how the amount of alcohol that you are consuming has changed?” | |
| --- | --- |
| Response: **I am drinking less alcohol**   - Supervised classification Label: **0** - Relative frequency: **13.9%**   - Gender:     - **female: 9.52%,**     - **male: 4.40%,**     - **Not prefer to say: 0.0%**   - Age:     - **2.20 %** in 20s     - **2.93 %** in 30s     - **3.66 %** in 40s     - **2.56 %** in 50s     - **1.46 %** in 60s     - **0.73 %** in 70s     - **0.37 %** in 80s | Response: **I am drinking more alcohol**   - Supervised classification Label: **1** - Relative frequency: **86.1%**   - Gender:     - **female: 70.33%,**     - **male: 15.38%,**     - **Not prefer to say: 0.37%**   - Age:     - **12.82 %** in 20s     - **29.67 %** in 30s     - **20.14 %** in 40s     - **13.55 %** in 50s     - **8.79 %** in 60s     - **1.10 %** in 70s     - **0.00 %** in 80s |

1. **Nonensemble methods**

We first tune the number of neighbors of the k-nearest neighbors (KNN) method, but the model performed poorly regardless of the number of neighbors that was taken into consideration during the learning process (see **Table 2** for the results of KNN with k=5). We then tune the number of hidden layers and nodes in each hidden layer of MLP using k-fold cross-validation on the training dataset, and it turns out that the most accurate MLP model is obtained with two hidden layers containing 5 nodes each (see **Table 2)**. We also apply decision trees with a maximum depth of 3, 4, and 5. It turns out that a decision tree with a maximum depth of 5 (DT-5) overfits the training data (train AUC of 0.99 and train accuracy of 98%), while a decision tree with a maximum depth of 3 (DT-3)does not overfit (train AUC 0.90 and train accuracy 94%), and its performance on the validations sets is acceptable (validation AUC of 0.7877 and validation accuracy of 91.92%). **Figure 1** demonstrates the AUROCs of DT-3 before and after SMOTE is applied.


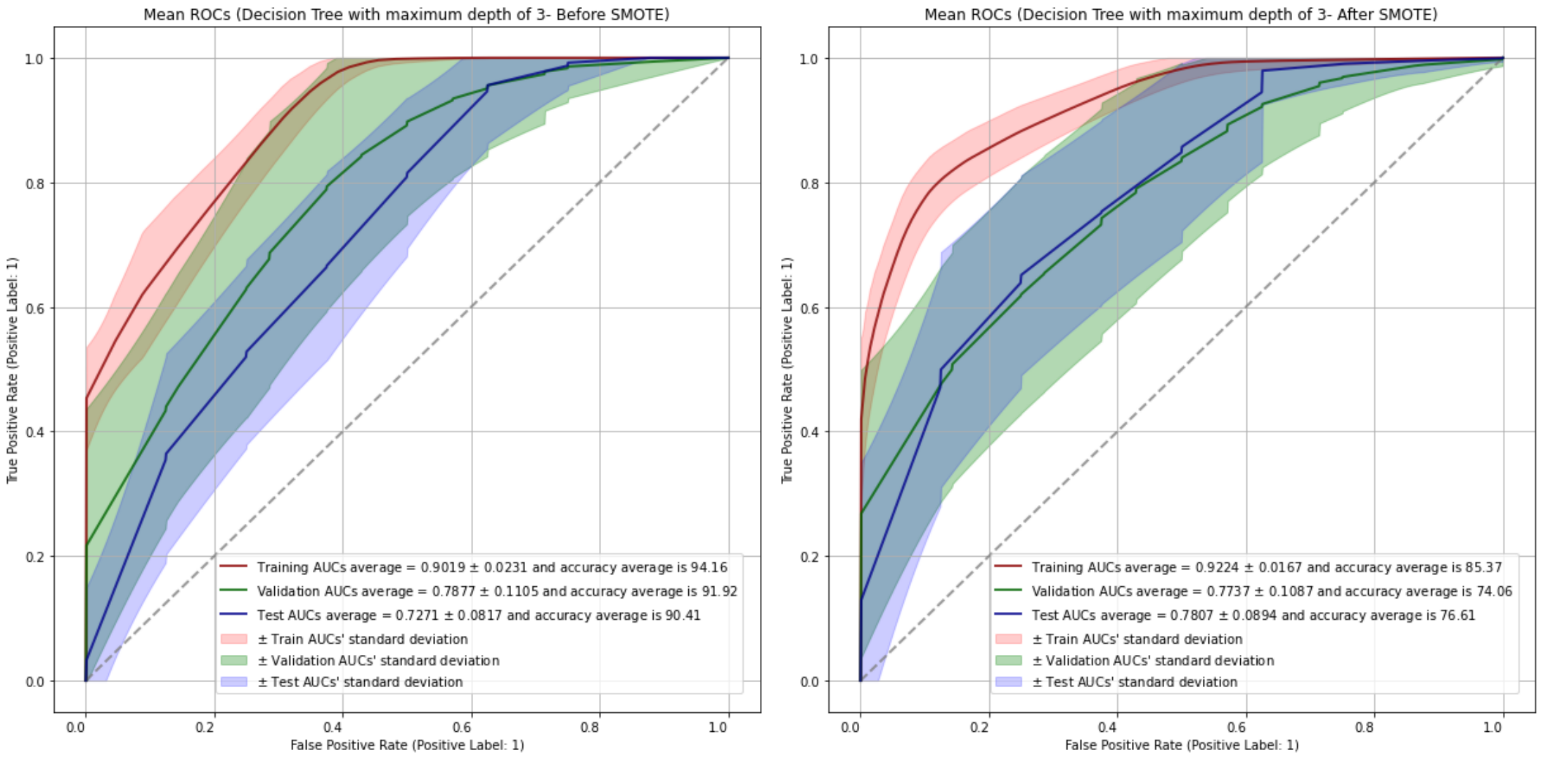


Figure 1. The performance of a decision tree with a maximum depth of 3 before and after SMOTE is applied.

For consistency purposes, we repeat the training-validation process for Logistic regression and SVM (linear kernel performs better than polynomial or Radial Basis Function) without tunning any hyperparameters. and MLP because their validation AUCs are the largest while they do not overfit the training data. Moreover, we apply SMOTE on the training sets and retrain all non-ensemble models to compare their performances before and after SMOTE is applied. **Table 2** displays the performances of all non-ensemble models on training, validation, and test sets before and after SMOTE is applied. Decision trees perform better than other non-tree-based models, and specifically a decision tree with a maximum depth of 3 does not overfit the training dataset.

Table 2. The performance of multi-layer perceptron with two hidden layers containing 5 nodes (MLP), support vector machines (SVM), logistic regression (LR), decision tree with maximum depth of 3 (DT-3), decision tree with maximum depth of 4 (DT-4), and decision tree with maximum depth of 5 (DT-5), before and after SMOTE is applied on training dataset (SMOTE was not applied on Validation or Test sets).

| **Model**  **Performance** | **KNN**  (Before SMOTE) | **MLP**  (Before SMOTE) | **SVM**  (Before SMOTE) | **LR**  (Before SMOTE) | **DT-3**  (Before SMOTE) | **DT-4**  (Before SMOTE) | **DT-5**  (Before SMOTE) |
| --- | --- | --- | --- | --- | --- | --- | --- |
| Train AUC | 0.8851 | 0.9658 | 0.8184 | 0.8782 | 0.9019 | 0.9767 | 0.9950 |
| Validation AUC | 0.6212 | 0.6034 | 0.6912 | 0.7217 | 0.7877 | 0.8828 | 0.8995 |
| Test AUC | 0.5187 | 0.7127 | 0.7771 | 0.8044 | 0.7271 | 0.7563 | 0.7900 |
| Train Accuracy | 87.33% | 95.92% | 63.01% | 91.37% | 94.16% | 95.13% | 97.92% |
| Validation Accuracy | 85.51% | 80.31% | 59.03% | 87.34% | 91.92% | 91.61% | 94.02% |
| Test Accuracy | 86.58% | 85.65% | 63.34% | 90.39% | 90.41% | 90.59% | 93.39% |
| **Model**  **Performance** | **KNN**  (After SMOTE) | **MLP**  (After SMOTE) | **SVM**  (After SMOTE) | **LR**  (After MOTE) | **DT-3**  (After MOTE) | **DT-4**  (After MOTE) | **DT-5**  (After SMOTE) |
| Train AUC | 0.9961 | 0.9553 | 0.9141 | 0.9496 | 0.9224 | 0.9647 | 0.9841 |
| Validation AUC | 0.6606 | 0.6327 | 0.6826 | 0.6725 | 0.7737 | 0.7820 | 0.8006 |
| Test AUC | 0.5056 | 0.7128 | 0.7301 | 0.7375 | 0.7807 | 0.8077 | 0.8242 |
| Train Accuracy | 91.50% | 91.74% | 79.36% | 88.38% | 85.37% | 91.69% | 94.80% |
| Validation Accuracy | 72.08% | 73.41% | 71.45% | 77.12% | 74.06% | 83.21% | 85.58% |
| Test Accuracy | 62.93% | 79.30% | 76.30% | 83.18% | 76.61% | 85.82% | 87.82% |

Moreover, logistic regression validation AUCs and accuracy scores are comparable with DT-3, and it does not overfit the training data. **Figure 2** displays the coefficients of the logistic regression when it is trained on the training data.


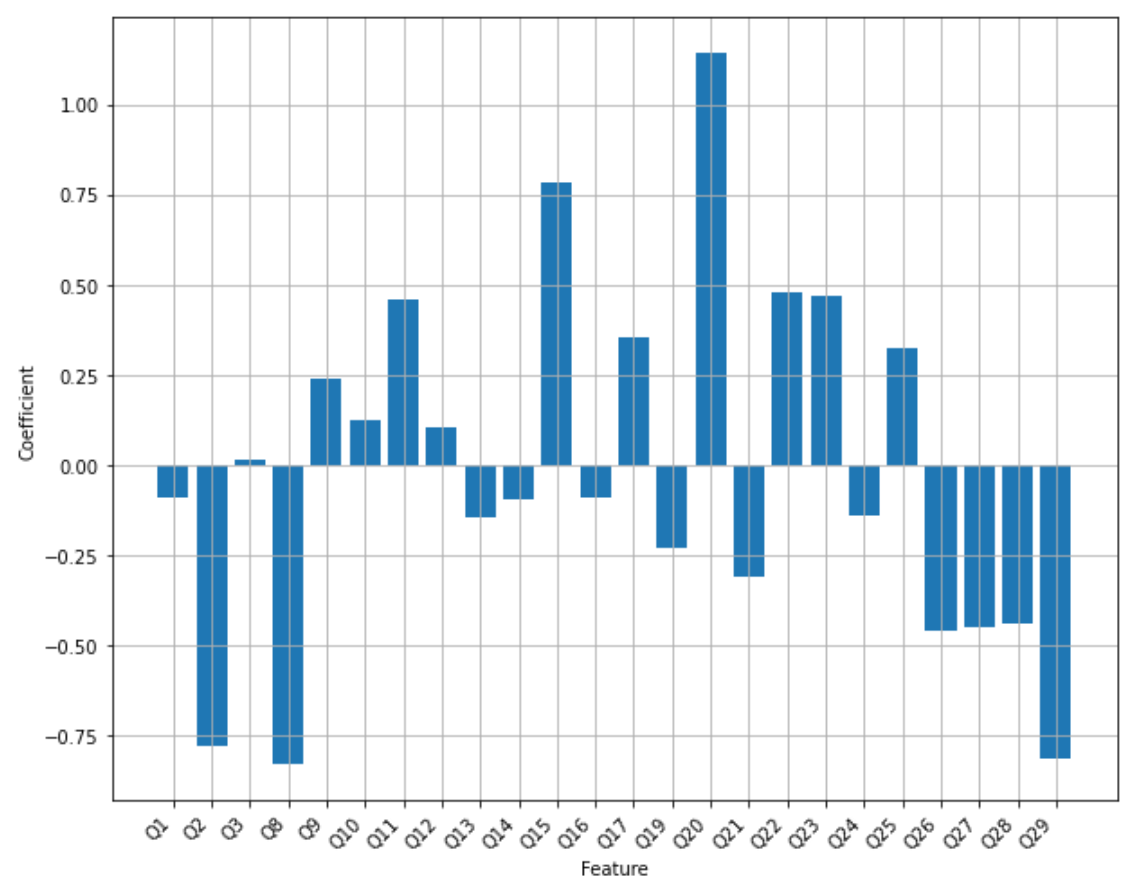


Figure 2. The logistic regression's coefficients before SMOTE is applied on the training set.

1. **Ensemble methods**

**Figure 3** compares the feature importance scores (percentage) of a random forest containing 4 trees of a maximum depth of 3 (RF-4) with a single decision tree with a maximum depth of 3 (DT-3).


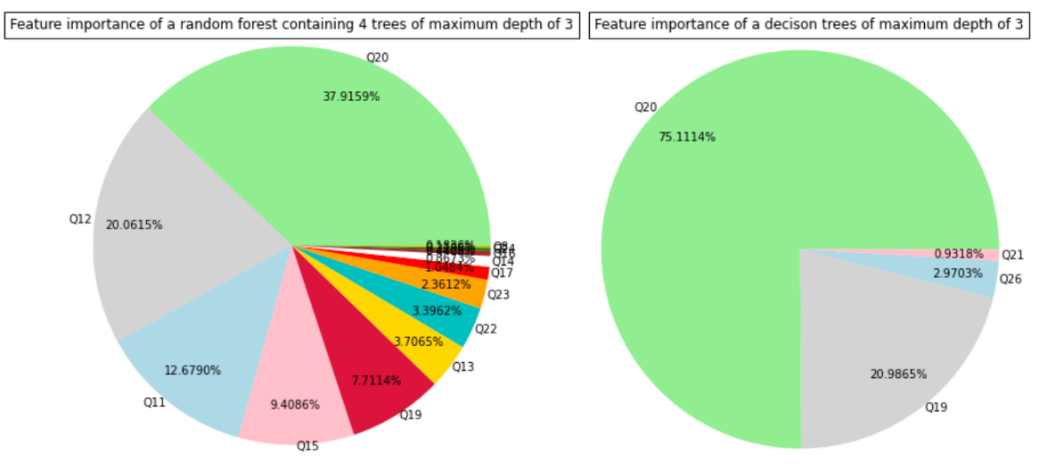


Figure 3. Feature importance scores (percentages) for a random forest with 4 trees of maximum depth of 3 (RF-4) in comparison to a single decision tree with maximum depth of 3 (DT-3).

The training test average of AUCs of AdaBoost with 8 stumps before and after SMOTE is applied to training sets are given in **Figure 4**.


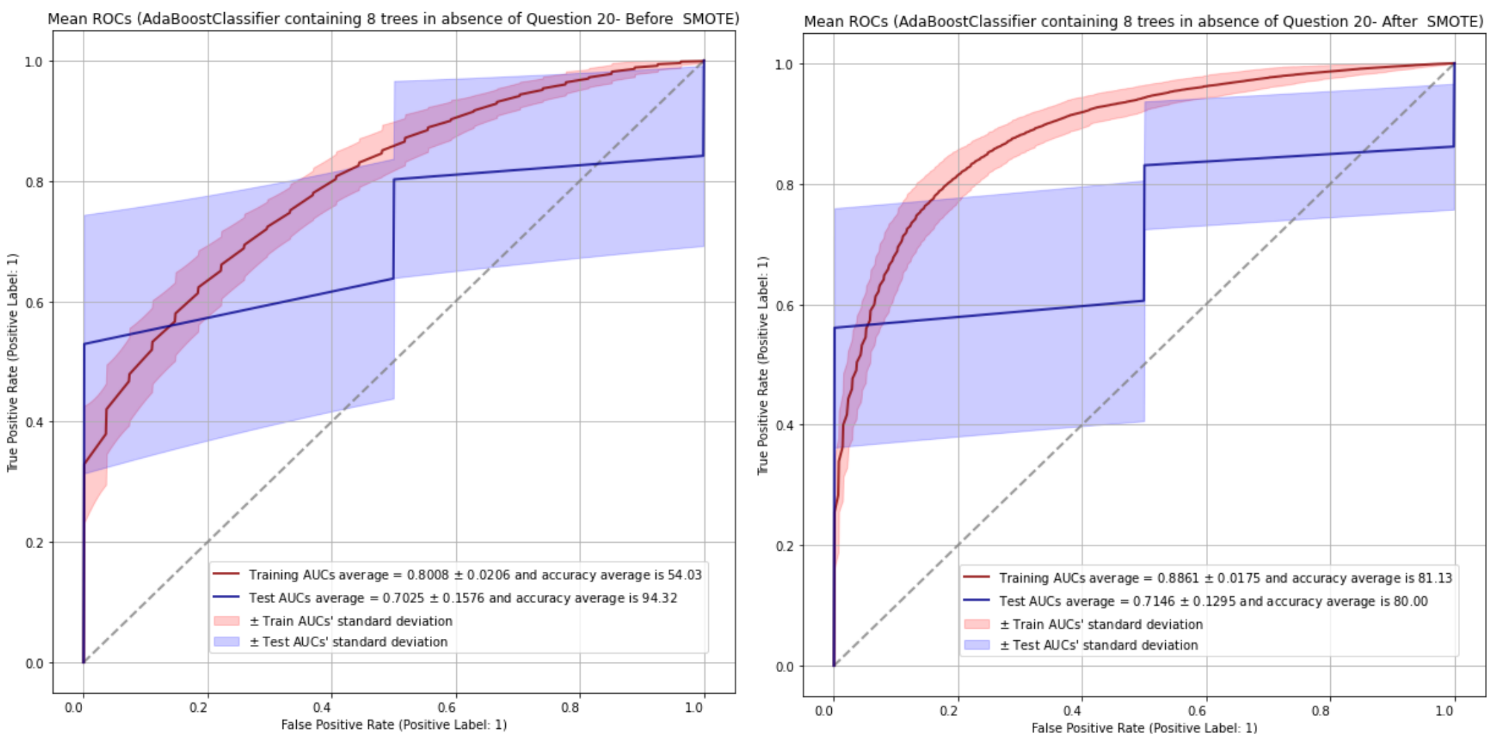


Figure 4. The AUROCs of AdaBoost with 8 stumps on training and test sets before and after SMOTE is applied on the training data.

# **References**

[1] Conroy, Deirdre, and Goldstein, Cathy. COVID Isolation on Sleep and Health in Healthcare Workers. Ann Arbor, MI: Inter-university Consortium for Political and Social Research [distributor], 2020-11-20. <https://doi.org/10.3886/E127081V1>
